# Supplementary material for: N-formyl-stabilizing quasi-catalytic species afford rapid and selective solvent-free amination of biomass-derived feedstocks
Source: Nat Commun. 2019 Feb 11;10:699. doi: 10.1038/s41467-019-08577-4 (PMC6370847; doi:10.1038/s41467-019-08577-4)
Supplement: Supplementary file 1 — Supplementary Information [file 41467_2019_8577_MOESM1_ESM.pdf]

<u>Supplementary Information</u>

N-formyl-stabilizing quasi-catalytic species afford rapid and selective solvent-free amination of biomass-derived feedstocks

Hu Li, et al.

Supplementary Methods

Materials:

Furfural (99%), formamide (≥99.5%), ammonium formate (≥99%), furfurylamine (≥99%), formic acid (≥96%), 5-(hydroxymethyl)furfural (98%), xylose (>99%), *N,N*-dimethylformamide (99.9%) *N*-methylformamide (>99%), fructose (99%), glucose (≥99.5%), sucrose (99.9%), cellobiose (≥98%), benzoic acid (99.5%), terephthalic acid (99%), isophthalic acid (99%), terephthalaldehyde (98%), 1,4-diacetylbenzene (99%), 4-carboxybenzaldehyde (98%), 2-acetylbenzoic acid (99%),4-oxocyclohexanecarboxylic acid (98%), 1,3-cyclohexanedione (98%), 3-oxo-cyclopentane-carboxylic acid (98%), valeric acid (99%), fumaric acid (99%), glutaraldehyde, succinaldehyde, 1,4-pentanedione, 2,5-hexanedione (97%), *o*-phthalaldehyde (>98%), 2-acetylbenzaldehyde (95%), 5-oxohexanoate (98), levulinic acid (98%), 4-benzoylbutyric acid (98%), 5-(4-methylphenyl)-5-oxovaleric acid (97%), 5-(3-fluorophenyl)-5-oxovaleric acid (97%), 4-(4-fluorobenzoyl)butyric acid (98%), 5-(2-fluorophenyl)-5-oxovaleric acid (97%), 5-cyclohexyl-5-oxovaleric acid (97%), 5-oxo-5-(2-thienyl)valeric acid (97%), 3-(2-oxo-cyclohexyl)-propionic acid, 2-carboxybenzaldehyde (98%), 2-acetylbenzoic acid (99%), succinic acid (99%), phthalic acid (99%), 3-methylphthalic acid, butyric acid (99%), 4-methyl-2-pentanone (99.5%), cellulose (Avicel® PH-101, average particle size 50 µm), SiC (~400 mesh particle size, ≥97.5%) and 1,3,5-trimethoxybenzene (99%) were purchased from Sigma-Aldrich (Tokyo or Shanghai). The 5-methylfurfural (98%), benzaldehyde (99%), veratraldehyde (98%), cinnamaldehyde (>99%), glycolaldehyde dimer (>95%), butyraldehyde (98%), 4-methyl-2-pentanone (99.5%), cyclopentanone (99%), cyclohexanone (99%), acetophenone (99%), cesium carbonate (99%), levulinic acid (98%), naphthalene (>99%), chloroform-d_1 (99.6 atom% D), tetrahydrofuran-d_8 (THF-d_8, 99.5 atom% D), formic acid-d_2 (95 wt% in D_2O, 98 atom% D), methyl formate (97%), and sodium periodate (99.8%), were supplied from Innochem Inc. (Beijing). Analytically pure solvents such as THF, acetone, methanol, *n*-hexane, acetonitrile, and ethyl acetate were provided by Aladdin Industrial Inc. (Shanghai). All chemicals were directly used

without further purification.

Synthesis of dialdehyde cellulose:

Dialdehyde cellulose was prepared by referring to a previously reported procedure with slight modification [1]. To a solution of 1 g sodium metaperiodate ($NaIO_4$) dissolved in 30 mL of deionized water, 1 g of cellulose was added. After stirring at room temperature for 48 h in the dark, the residue periodate was removed by decomposition with excess ethylene glycol (10 mL). The remaining solid was separated out by centrifugation, washing with water (20 mL × 5), and finally drying at 60 ºC overnight to give the product. Product structure was identified with a FT-IR on a Perkin-Elmer 1710 spectrometer, and its nitrogen content was determined by elemental analyses (Vario EL III, Elementar).

Supplementary Table 1 Microwave-assisted amination of furfural (FUR) with reactants formic acid (FA) and formamide (AM) showing product yield and carbon balance (CB)

Entry	Reactant(mmol)			Temp. (ºC)	Time (min)	FUR Conv. (%)	Product yield (%)				CB (%)
	FUR	FA	AM				**FDFAM**	**1**	**2**	**3**	
1	2	-	10	180	10	43	32	0	0	0	89
2[a]	2	-	10	160	10	87	63	0	0	0	76
3	2	6	10	200	3	100	<1	42	52	2	97
4	2	6	4	200	3	85	2	13	67	<1	97
5	2	2	10	200	3	76	16	43	15	<1	98

[a] Amberlyst-15 was added.

FDFAM: *N,N'*-(furan-2-ylmethylene)diformamide; **1:** *N*-(furan-2-ylmethyl)formamide; **2:** *N,N'*-bis(furan-2-ylmethyl)formamide; **3:** tris(furan-2-ylmethyl)amine

Supplementary Table 2 Rate constants of furfural (FUR) amination with formamide (AM) and formic acid (FA) at different temperatures.

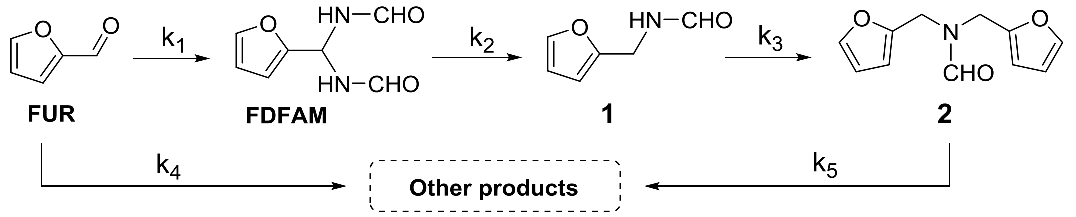

Entry	Temp(ºC)	Rate constants (min^{-1}) [b]				
		k_1	k_2	k_3	k_4	k_5
1	120	0.152	0.021	0.007	0.012	0
2	140	0.353	0.162	0.027	0.017	0.080
3	160	0.489	0.681	0.013	0.008	0.032
4	180	1.121	2.279	0.090	0.011	0.022
5	200	2.109	7.778	0.322	0.048	0.045
6 [a]	180	0.026	0.951	0.004	0.001	0.003

Reaction conditions: 2 mmol FUR, 6 mmol FA, 10 mmol AM, (120 to 200) ºC, (1 to 30) min reaction time with microwave irradiation.

[a] Oil bath heating. [b] Rate constants obtained by fitting rate data (Fig. 2 and Supplementary Fig. 3) and assuming pseudo first-order reaction kinetics.

Supplementary Table 3 Octanol-water partition coefficient (Log*P*) values for reactants and products estimated with an online source [a]

Entry	Structure	Name	Log*P*
1	$HCOONH_4$	Ammonium Formate (AMF)	<-1
2	$HCONH_2$	Formamide (AM)	-0.89
3	HCOOH	Formic acid (FA)	-0.51
4	H_2O	Water	-0.29
5	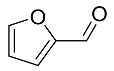	Furfural (FUR)	0.98
6	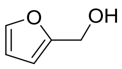	Furfuryl alcohol (FFA)	0.53
7	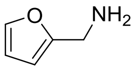	Furfurylamine (FAM, **4**)	0.38
8	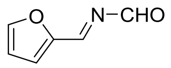	*N*-(Furan-2-ylmethylene)formamide	0.41
9	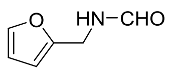	*N*-(Furan-2-ylmethyl)formamide (FFAM, **1**)	0.14
10	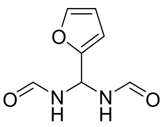	*N,N'*-(Furan-2-ylmethylene)diformamide (FDFAM)	-0.52
11	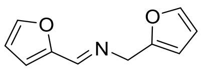	1-(Furan-2-yl)-*N*-(furan-2-ylmethyl)methanimine	1.69
12	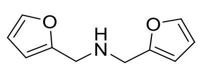	Bis(furan-2-ylmethyl)amine (BFAM, **5**)	1.41
13	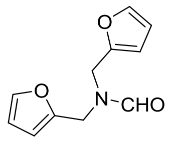	*N*-(Furan-2-ylmethyl)formamide (BFFAM, **2**)	1.04
14	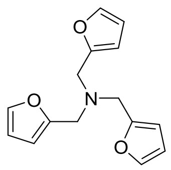	Tris(furan-2-ylmethyl)amine (TFFAM, **3**)	2.31

[a] Log*P* was calculated with the JSME Molecular Editor developed by Peter Ertl and Bruno Bienfai: http://www.molinspiration.com/cgi-bin/properties (Access on June 10, 2018)

Supplementary Table 4. Control of microwave heating parameters in the amination of FUR and AM using FA. Reaction conditions: 2 mmol FUR, 12 mmol AM, 6 mmol FA, 180 ºC.

Entry	Reactor type [a]	SiC powder [b]	Initial heating time (s)	Reaction time (min)	**1** yield (%)	Carbon
1	Microwave reactor used in this work	Not used	~30	3	98	Trace
2	Microwave reactor used in this work	Used	~22	2.5	94	Smack
3	Microwave reactor used in this work	Used	~22	3	91	Smack
4	Microwave reactor used in this work	Used	~22	5	72	Plenty
5	Oil-bath	Not used	~380	60	69	Smack
6	Oil-bath	Used	~378	60	70	Smack
7	Anton Paar Microwave 300	Not used	~65	3	95	Trace

[a] The maximum microwave power value is the only difference between the microwave reactor (700 W) used in this work and Anton Paar Microwave 300 (300 W; Graz, Austria), which affects the initial heating rate to reach the set temperature (180 ºC); However, only (25 to 30) W was required to keep the specific temperature in the reaction.
[b] 0.1 g SiC powder was added together with the reactants.

With the addition of SiC powder, which completely absorbs microwave energy, primarily convective heating occurs and for a reaction mixture of FUR, AM, and FA (Supplementary Table 4), the reaction rate can be further accelerated (reaction completion within ca. 2.5 min, shorter than 3 min) despite forming a certain amount of carbon (entries 2 & 3), which affirms the positive role of microwave in the reaction process. With prolonging the reaction time from 2.5 min to 3 min and to 5 min (entries 3-4), the carbon content further increased while the yield of **1** decreased significantly, implying that overheating at 180 ºC caused by hotspots on the SiC powder may lead to the occurrence of side reactions like condensation to give carbon in the reaction period. However, the overheating phenomenon was not detected in the oil-bath heating system (entries 5 & 6), indicating that microwave is the rapid heating source in the reaction process. In addition, comparable yields of **1** obtained using Anton Paar Microwave reactor with a lower maximum microwave power of 300 W (entry 7 versus entry 1) were obtained, where relatively longer reaction times were required to reach the set temperature (180 ºC) in comparison with the microwave reactor (700 W) used in this work. This result, on the other hand, further illustrates that side reactions mainly took place at high temperatures along with overheating during the reaction other than the heating process at a relatively low (<180 ºC) temperature range.

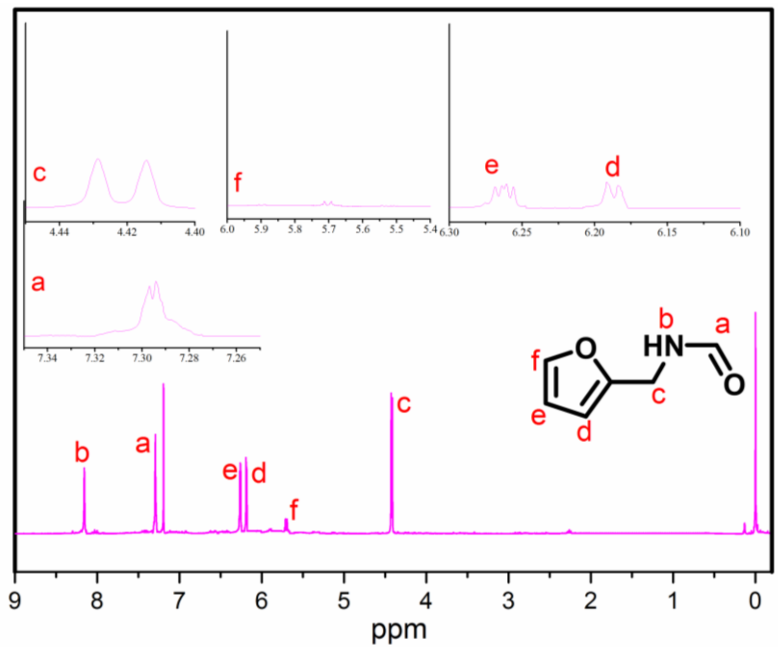

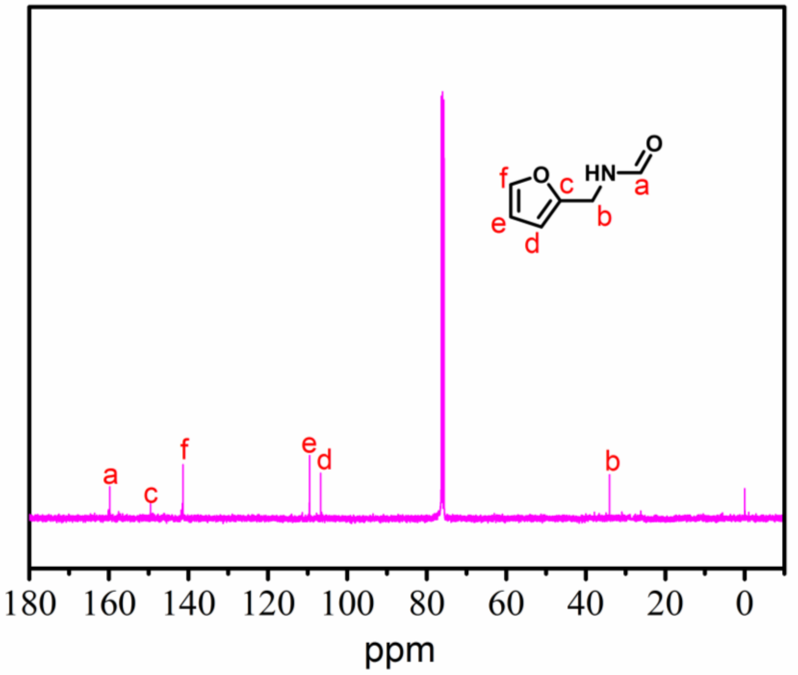

Supplementary Figure 1 ^{1}H and ^{13}C NMR spectra of isolated products, *N*-(furan-2-ylmethyl) formamide (**1**). TMS and $CDCl_3$ were used as internal standard and solvent, respectively.

^{1}H NMR (400 MHz, $CDCl_3$): δ 8.16 (s, 1H), 7.29 (d, J = 0.7 Hz, 1H), 6.26 (dd, J = 3.1, 1.9 Hz, 1H), 6.19 (d, J = 3.2 Hz, 1H), 5.66 (s, 1H), 4.42 (d, 2H).

^{13}C NMR (125 MHz, $CDCl_3$): δ 159.78 (s), 149.54 (s), 141.36 (s), 109.50 (s), 106.72 (s), 34.03 (s).

HRMS (m/z): $[M]^+$ calcd. for $C_6H_7NO_2$, 125.0477; found, 125.0486

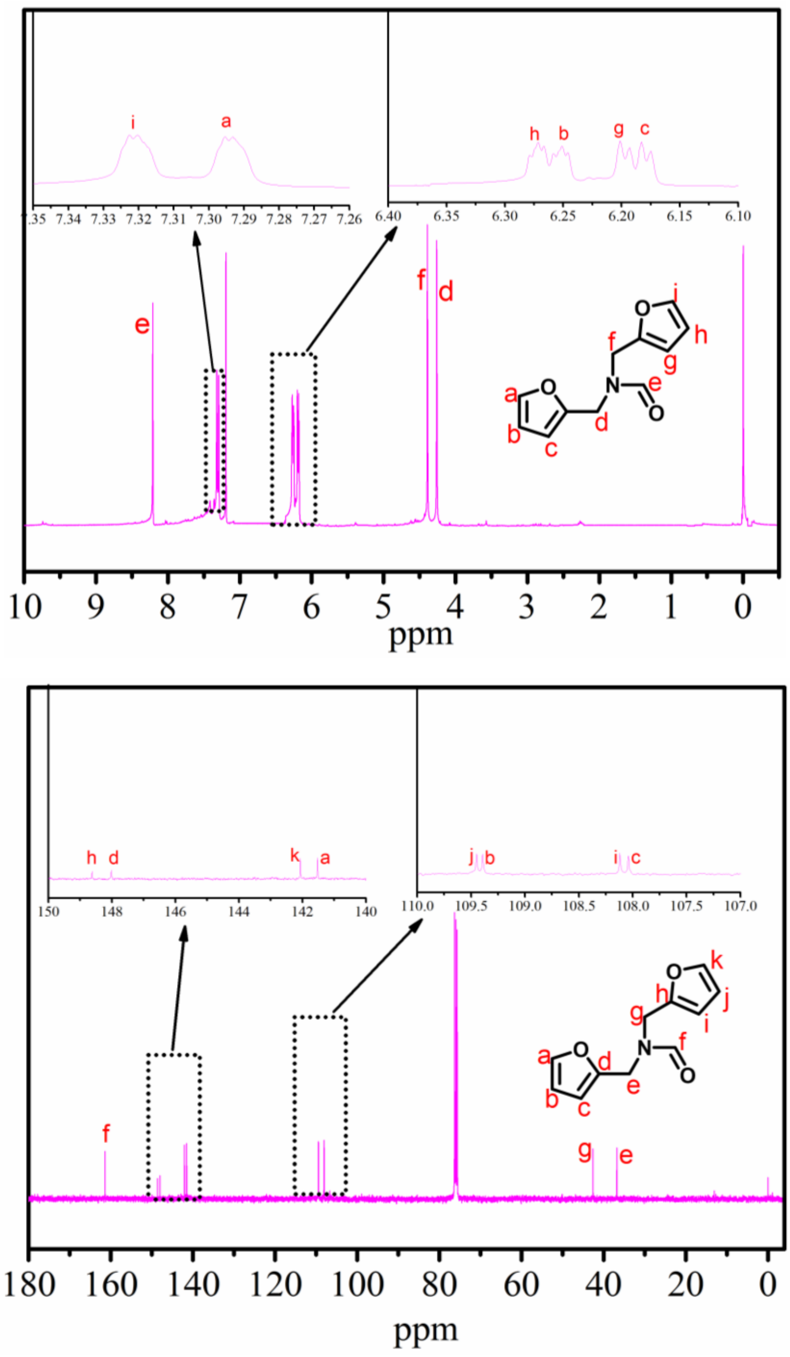

Supplementary Figure 2 ^{1}H and ^{13}C NMR spectra of isolated products, *N,N*-bis(furan-2-ylmethyl) formamide (**2**). TMS and $CDCl_3$ were used as internal standard and solvent, respectively.

^{1}H NMR (400 MHz, $CDCl_3$): δ 8.21 (s, 1H), 7.31 (d, J = 10.9 Hz, 2H), 6.26 (d, J = 8.2 Hz, 2H), 6.19 (d, J = 7.3 Hz, 2H), 4.39 (s, 2H), 4.26 (s, 2H)

^{13}C NMR (125 MHz, $CDCl_3$): δ 161.40 (s), 148.62 (s), 148.02 (s), 142.06 (s), 141.52 (s), 109.42 (d, J = 5.7 Hz), 108.08 (d, J = 8.0 Hz), 42.62 (s), 36.82 (s)

HRMS (m/z): $[M]^+$ calcd. for $C_{11}H_{11}NO_3$, 205.0739 ; found, 205.0745

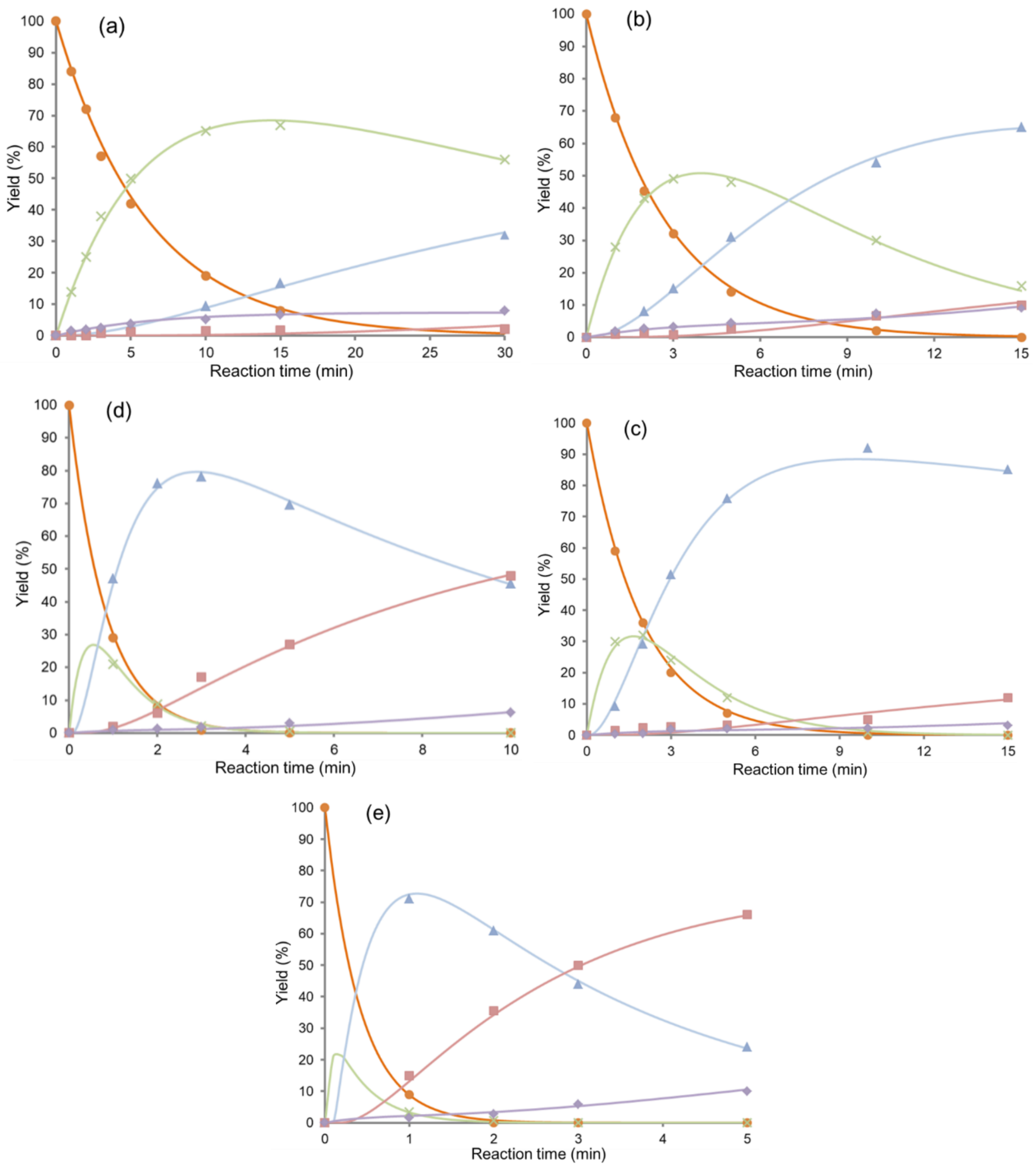

Supplementary Figure 3 Kinetic rate data of microwave-assisted amination of furfural (FUR) with formamide (AM) at temperatures: (a) 120 ºC, (b) 140 ºC, (c) 160 ºC, (d) 180 ºC, and (e) 200 ºC. Rate data modeled by assuming pseudo first-order reaction of all species (● FUR, × FDFAM, ▲ **1**, ■ **2**, ◆ Other). Reaction conditions: 2 mmol FUR, 6 mmol FA, and 10 mmol AM. Products: FDFAM: *N,N'*-(Furan-2-ylmethylene)diformamide; **1**: *N*-(Furan-2-ylmethyl) formamide; **2**: *N,N'*-Bis(furan-2-ylmethyl) formamide

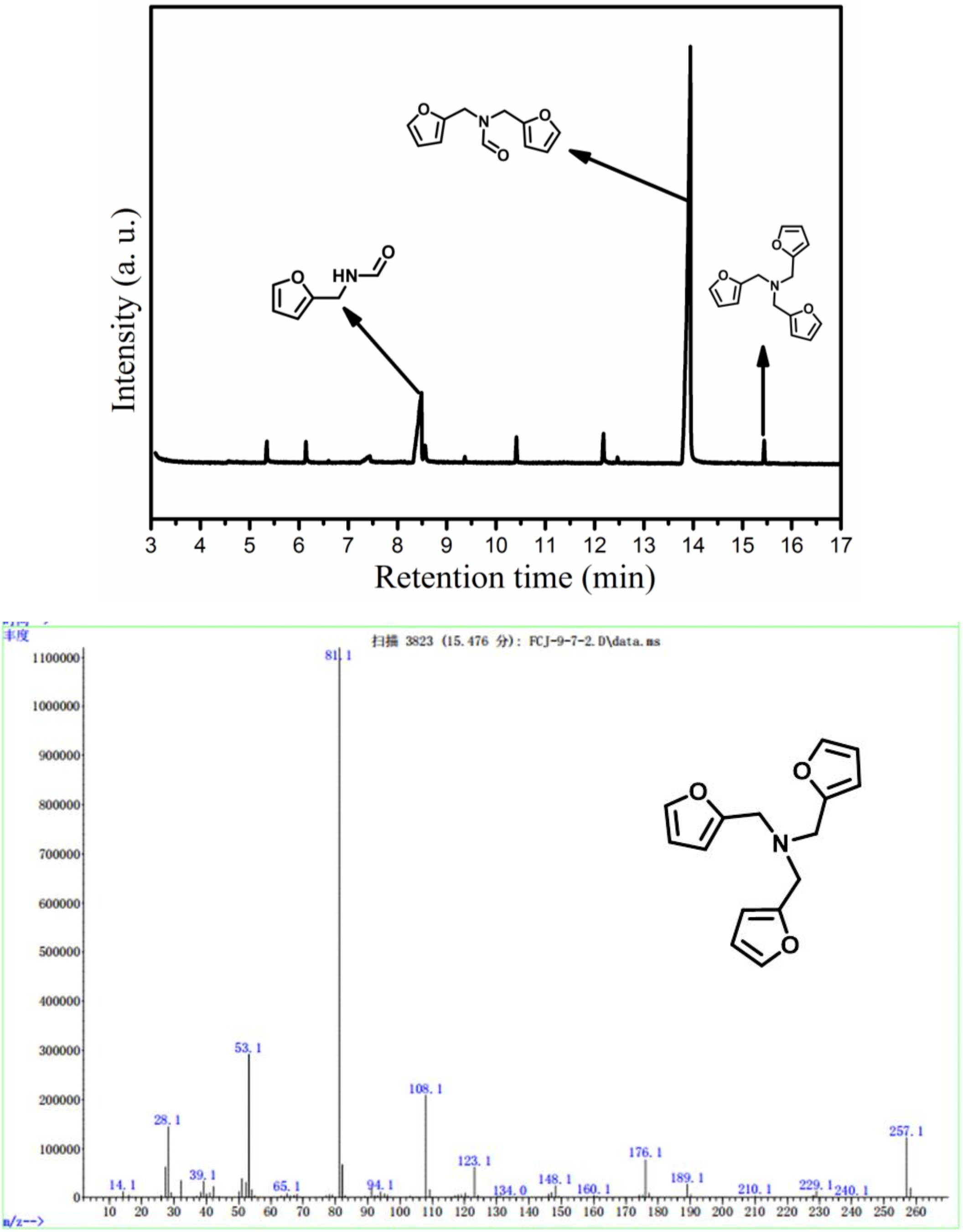

Supplementary Figure 4 GC-MS spectrum of reaction mixture diluted by THF after microwave-assisted reaction of FUR (2 mmol), AM (10 mmol), and FA (6 mmol) at 200 ºC for 5 min; abundance versus m/z, scan (3823), 15.476 min

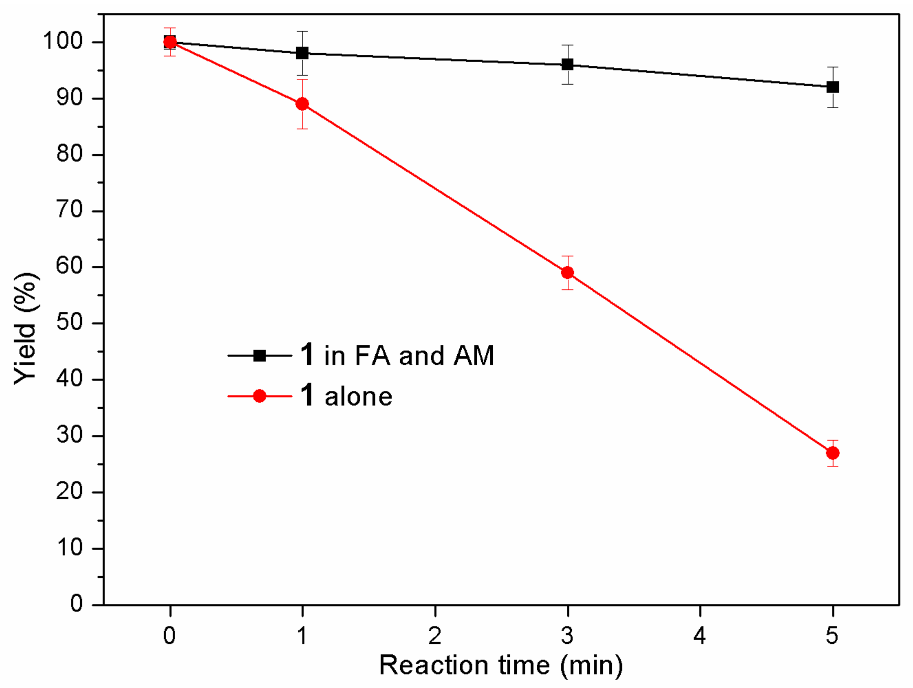

Supplementary Figure 5 Stability of *N*-(Furan-2-ylmethyl) formamide (**1**) in formic acid (FA) and formamide (AM) or alone under microwave irradiation; Reaction conditions: 2 mmol **1**, 6 mmol FA, 10 mmol AM, 160 ºC. Error bars with standard deviation (σ) of ≤3.8%.

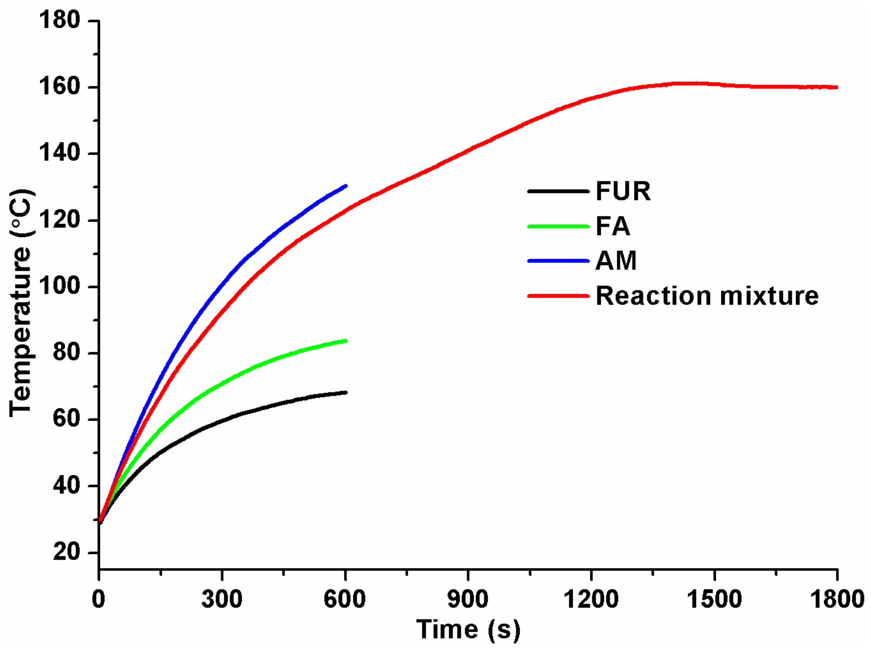

Supplementary Figure 6 Temperature-time profiles for 3 mL of reaction mixture and reactants (FUR, FA, AM) under microwave irradiation with 30 W constant power for microwave reactor of this work.

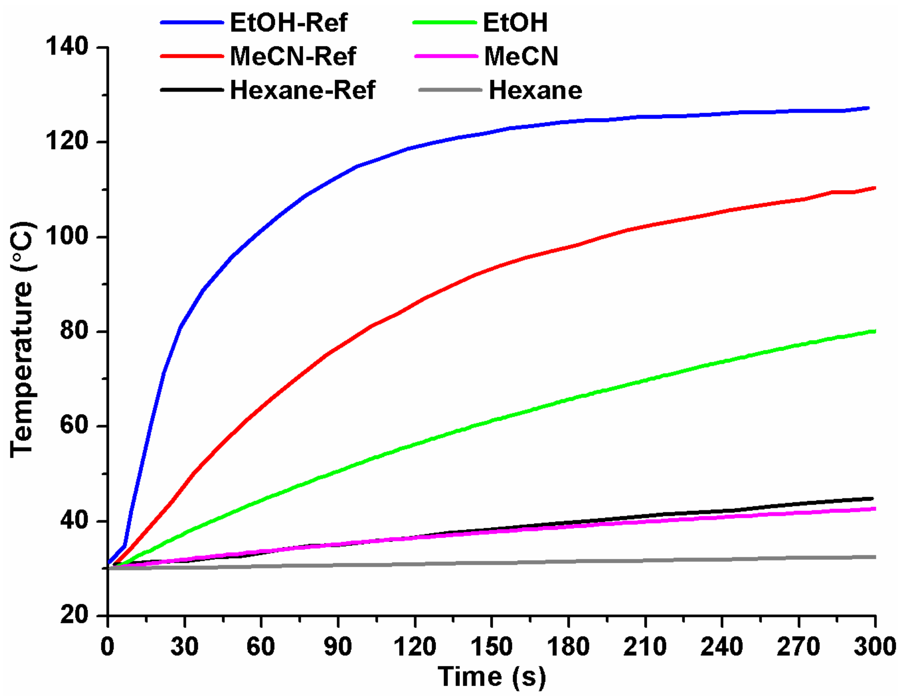

Supplementary Figure 7 Temperature-time profiles for 3 mL of EtOH, MeCN and hexane for microwave reactor of this work.

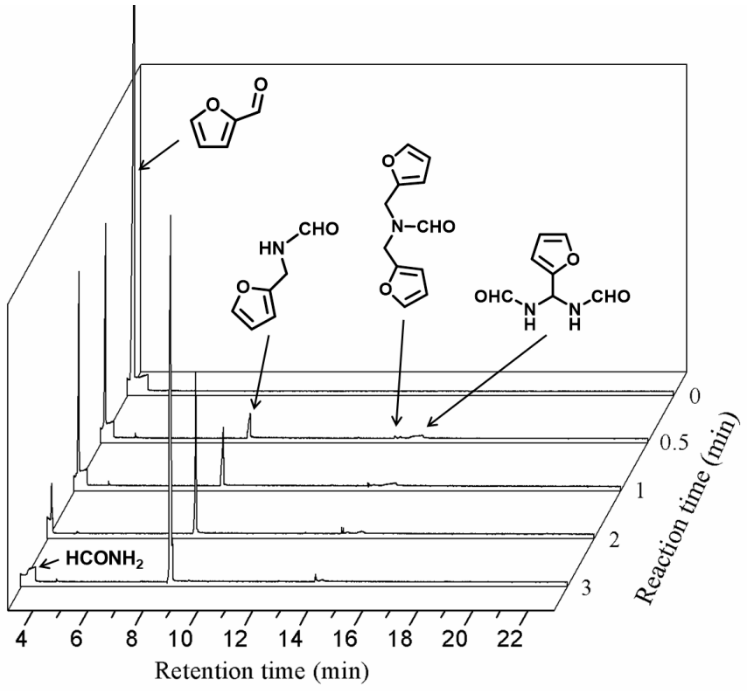

Supplementary Figure 8 Typical GC spectra of product solution diluted with THF obtained from microwave-assisted reaction after varying reaction time. Reaction conditions: FUR (2 mmol), AM (12 mmol), FA (6 mmol), 180 ºC. Corresponding MS spectra are provided in Supplementary Figures 9-13.

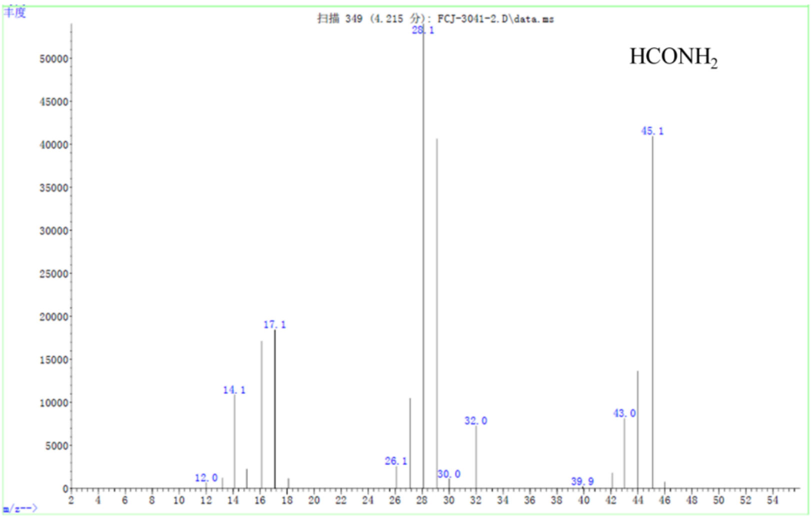

Supplementary Figure 9 MS spectrum of AM. Abundance versus m/z, scan (349) at 4.215 min

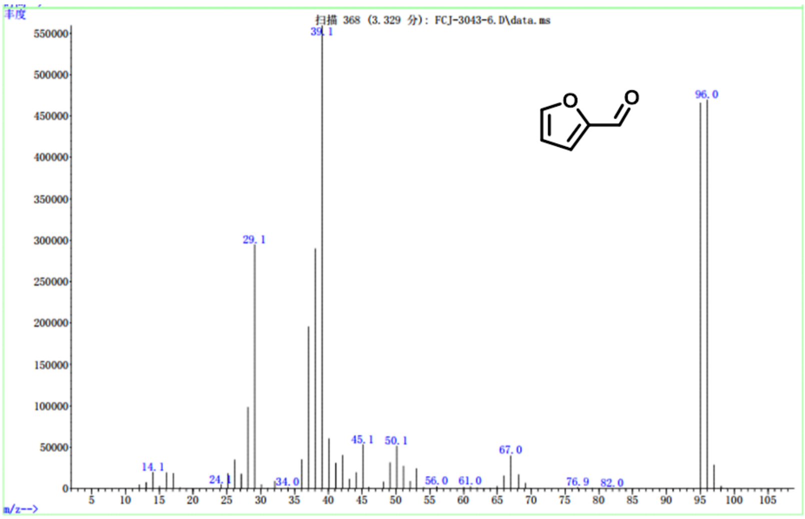

Supplementary Figure 10 MS spectrum of FUR. Abundance versus m/z, scan (368) at 3.329 min

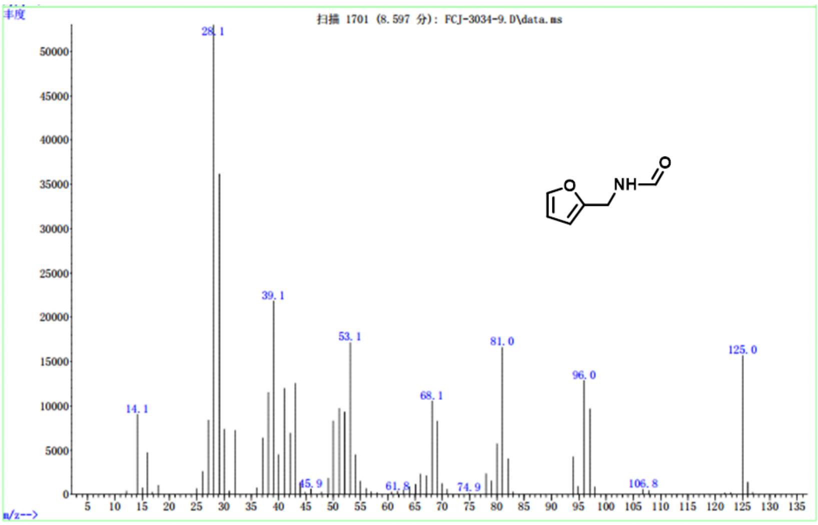

Supplementary Figure 11 MS spectrum of **1**. Abundance versus m/z, scan (1701) at 8.597 min

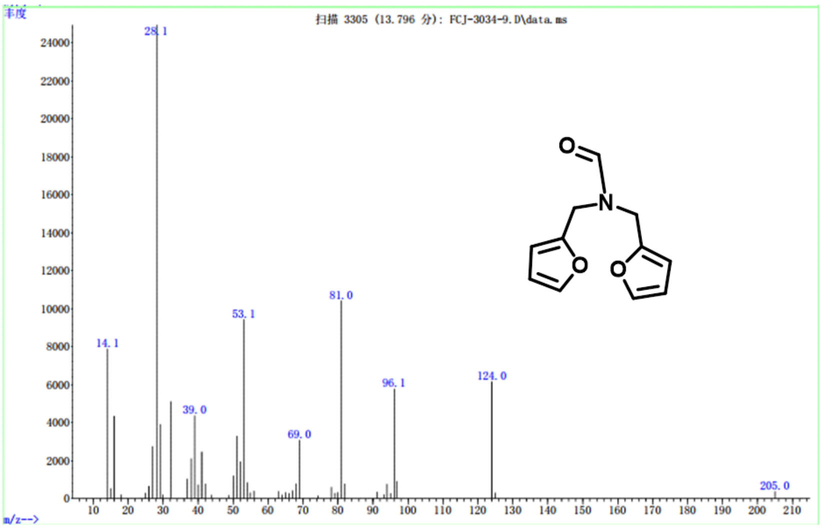

Supplementary Figure 12 MS spectrum of **2**. Abundance versus m/z, scan (3305) at 13.796 min

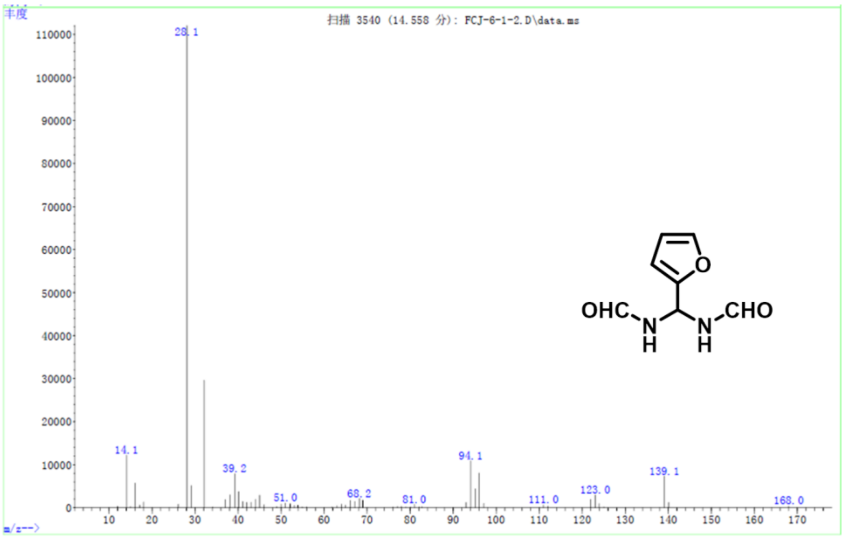

Supplementary Figure 13 MS spectrum of FDFAM. Abundance versus m/z, scan (3540) at 14.550 min

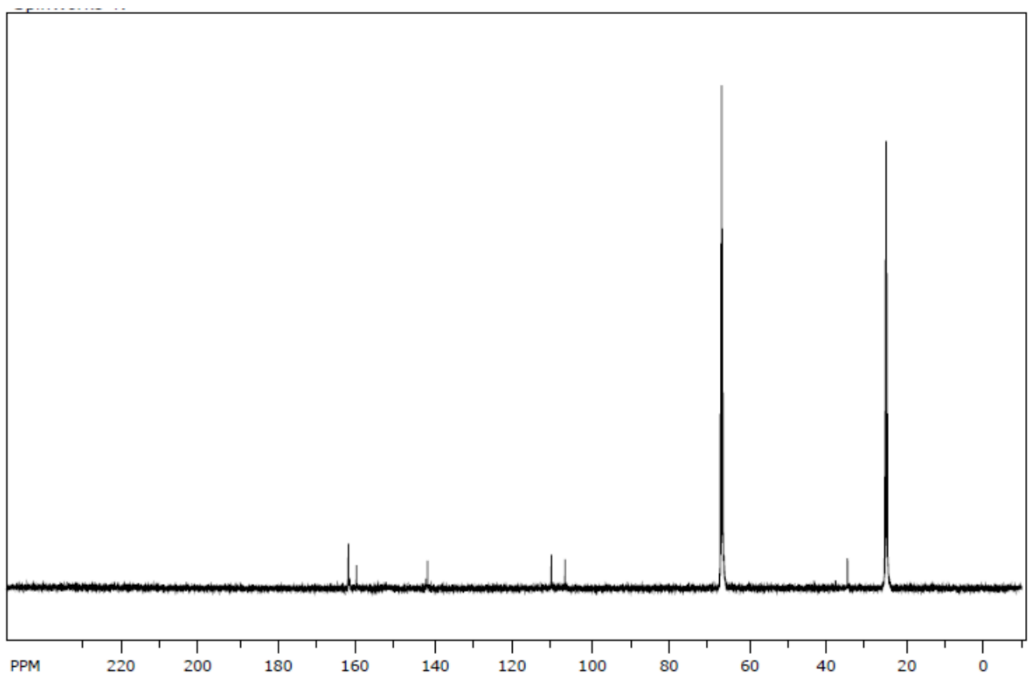

Supplementary Figure 14 ^{13}C NMR spectrum of product solution diluted by THF-d_8 obtained under microwave irradiation. Reaction conditions: FUR (2 mmol), AM (12 mmol), and FA (6 mmol), 180 ºC, 3 min. Reaction products are AM and **1**. No other products are evident, by referring to the ^{13}C NMR spectrum of isolated **1** in Supplementary Figure 1.

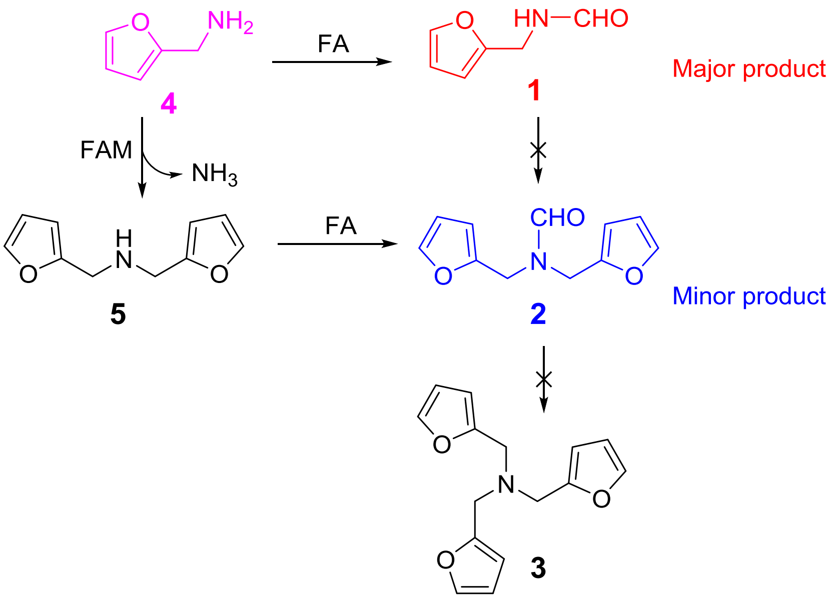

Supplementary Figure 15 Schematic of products obtained from reaction of **4** with FA under microwave irradiation. Reaction conditions: **4** (2 mmol), FA (6 mmol), 180 ºC, 3 min

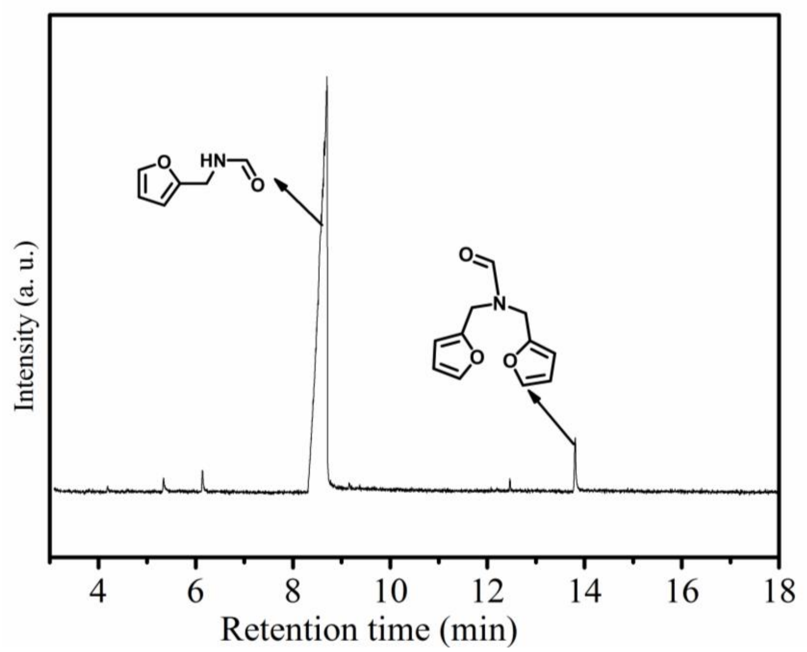

Supplementary Figure 16 GC-MS spectrum of product solution diluted by THF obtained from microwave irradiation of **4** (2 mmol) and FA (15 mmol) at 180 ºC for 3 min; Products **1** (ca. 85% yield) and **2** (ca. 10% yield) were observed.

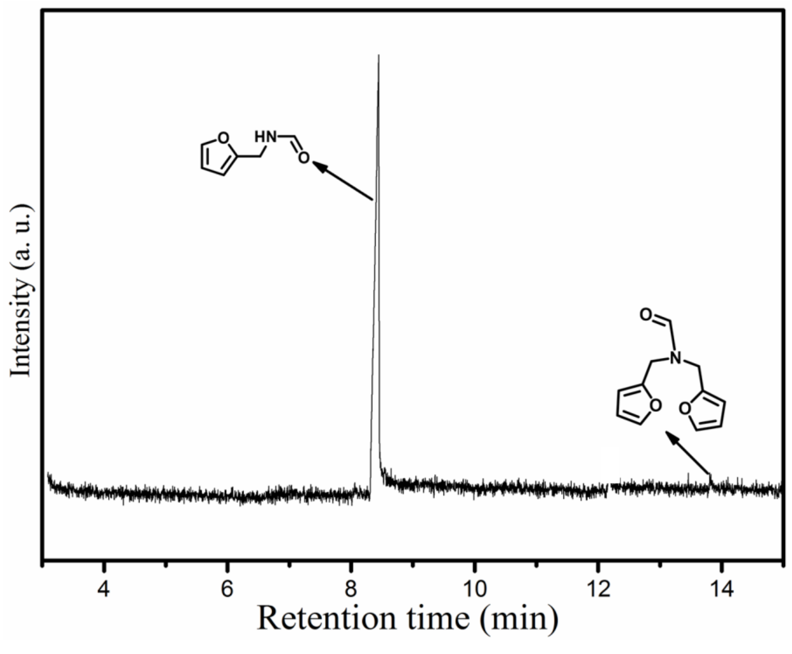

Supplementary Figure 17 GC-MS spectrum of product solution diluted by THF obtained from microwave-assisted reaction of **1** (2 mmol), AM (10 mmol), and FA (6 mmol) at 180 ºC for 3 min; Products FFAM and **2** (trace) were observed.

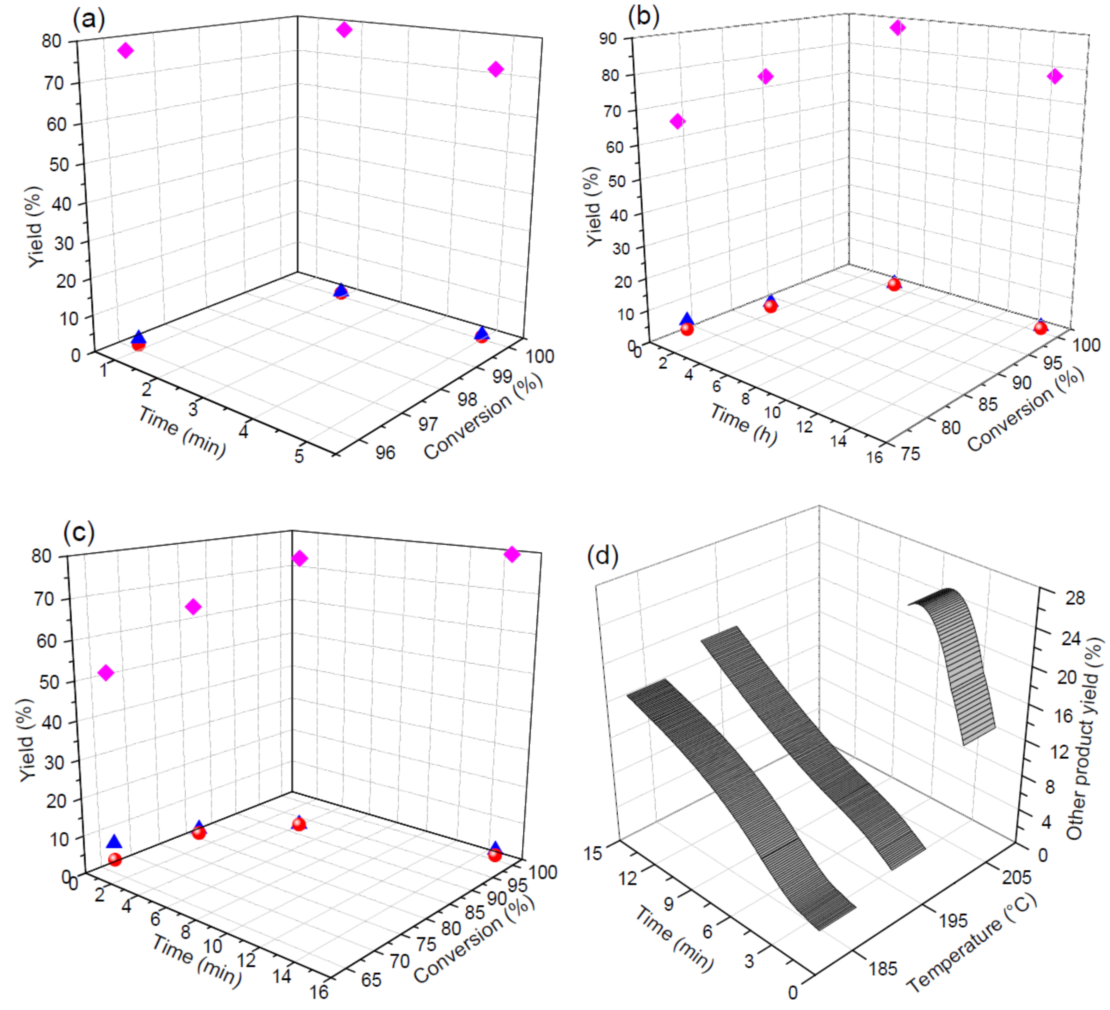

Supplementary Figure 18 Microwave-assisted amination of furfural (FUR) with ammonium formate (AMF) showing ● **1**, ▲ **2**, ♦ **3** at: (a) 205 ºC, (b) 195 ºC, (c) 185 ºC and (d) all temperatures for other products.

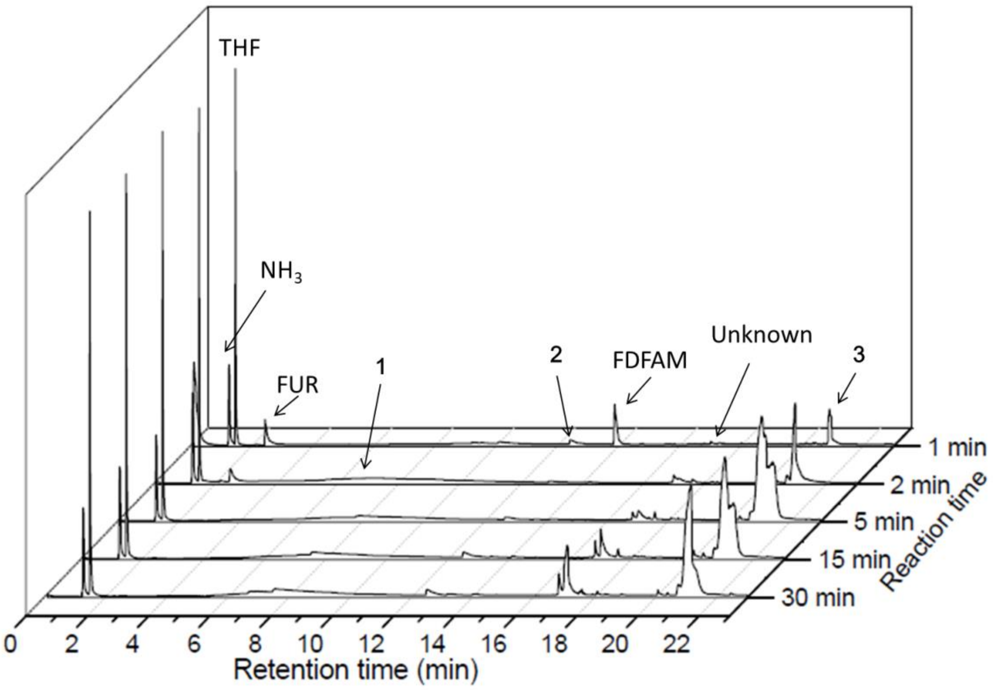

Supplementary Figure 19 GC spectra of product solutions diluted by THF obtained from microwave irradiation for different reaction times (along with MS spectrum of FDFAM); Reaction conditions: FUR (2 mmol), AMF (12 mmol), 195 ºC. The broad peak occurring at retention times of 6 min to 11 min is due to other products included with **1** and a certain amount of AM.

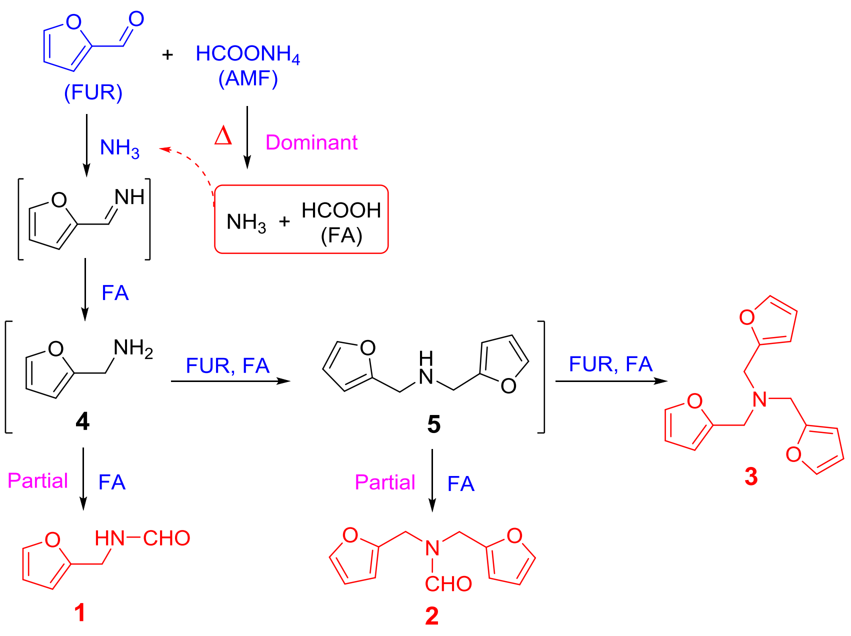

Supplementary Figure 20 Schematic illustration of reaction between furfural (FUR) and ammonium formate (AMF)

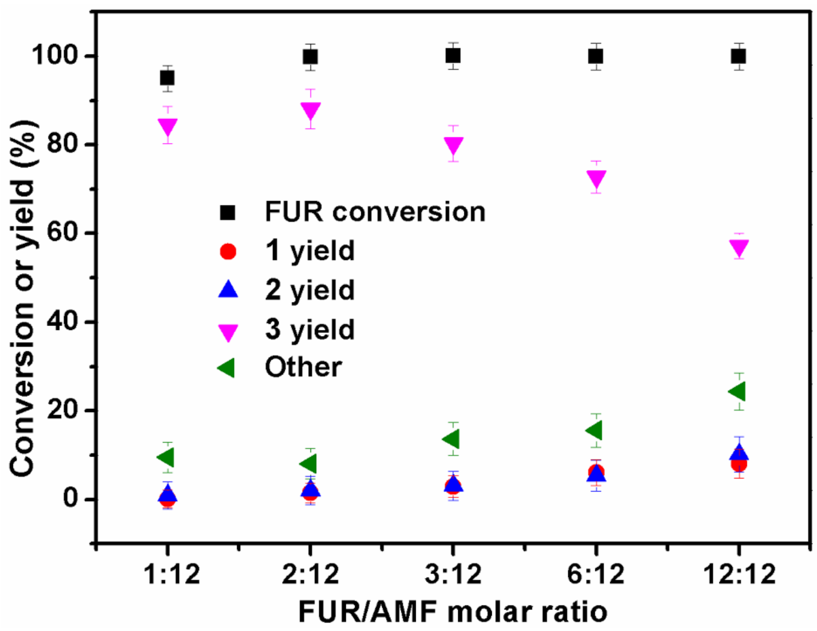

Supplementary Figure 21 Variation of furfural conversion and product (**1**, **2** and **3**) yields with furfural/ammonium formate (FUR/AMF) molar ratios obtained from microwave irradiation of solutions; reaction conditions: FUR fixed at 2 mmol, 195 ºC, 5 min. Error bars with standard deviation (σ) of ≤1.8%.

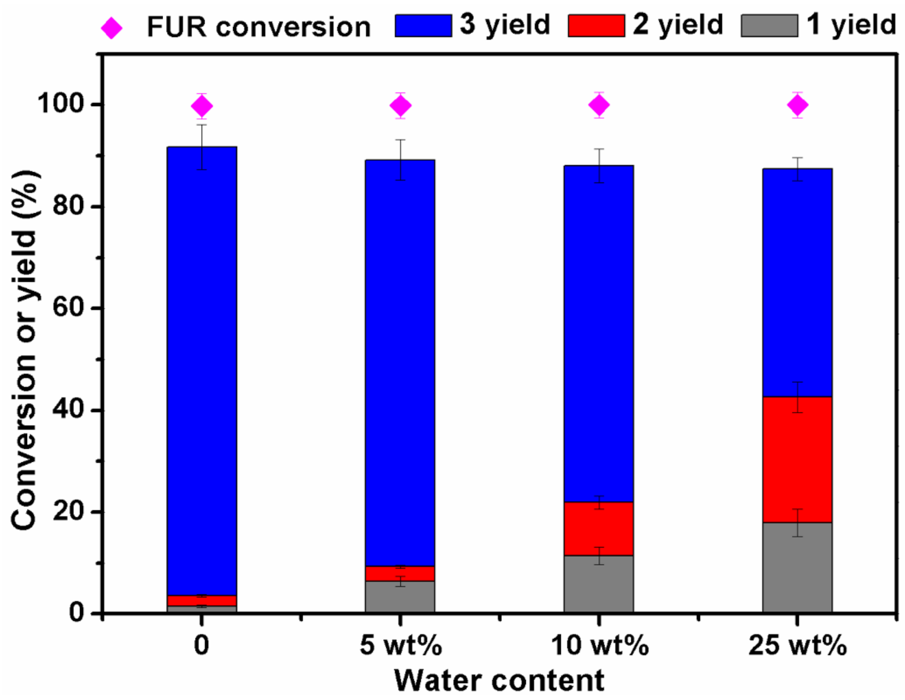

Supplementary Figure 22 Effect of water content on the amination of furfural (FUR) and ammonium formate (AMF) to give product **1**, **2** and **3**; Reaction conditions: FUR (2 mmol), AMF (12 mmol), 195 ºC, 5 min microwave irradiation. Error bars with standard deviation (σ) of ≤3.3%.

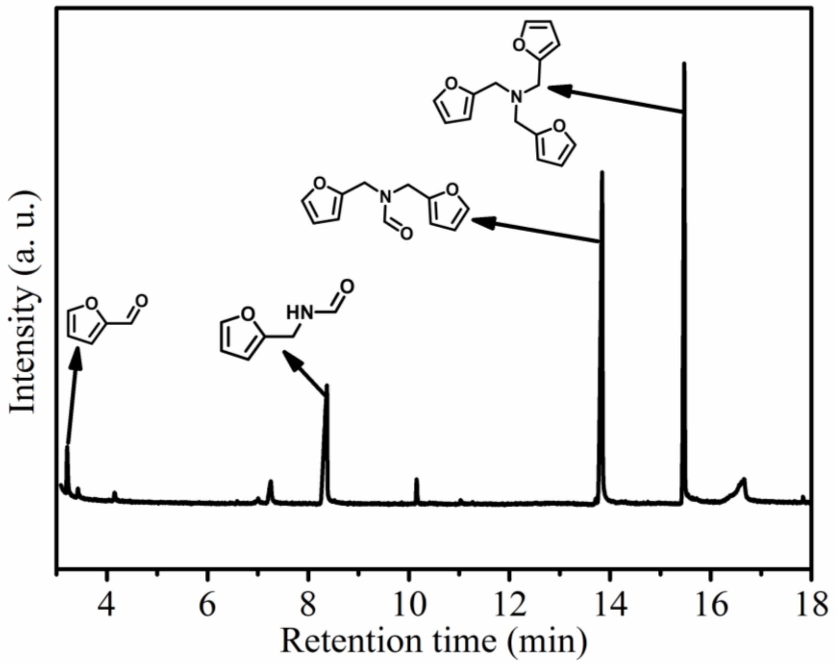

Supplementary Figure 23 GC-MS spectrum of product solution diluted by THF obtained from microwave irradiation of furfural (FUR) (2 mmol) and ammonium formate (AMF) (12 mmol) with 25 wt% water relative to FUR at 195 ºC for 3 min.

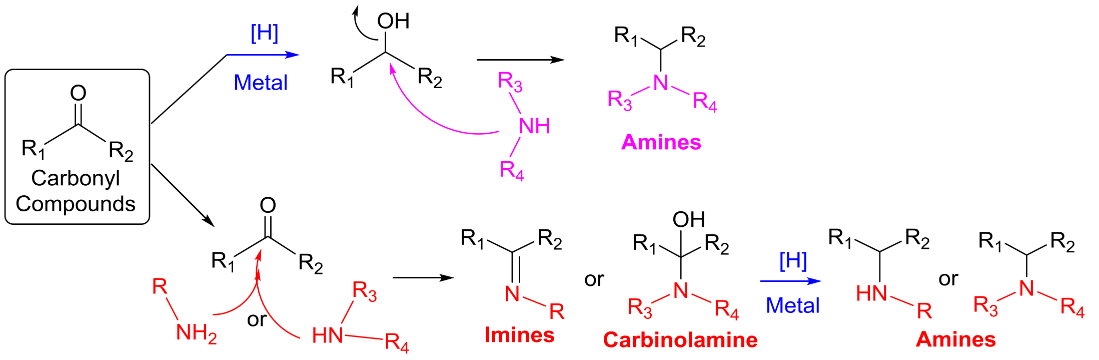

Supplementary Figure 24 Schematic illustration of reaction pathways for reductive amination

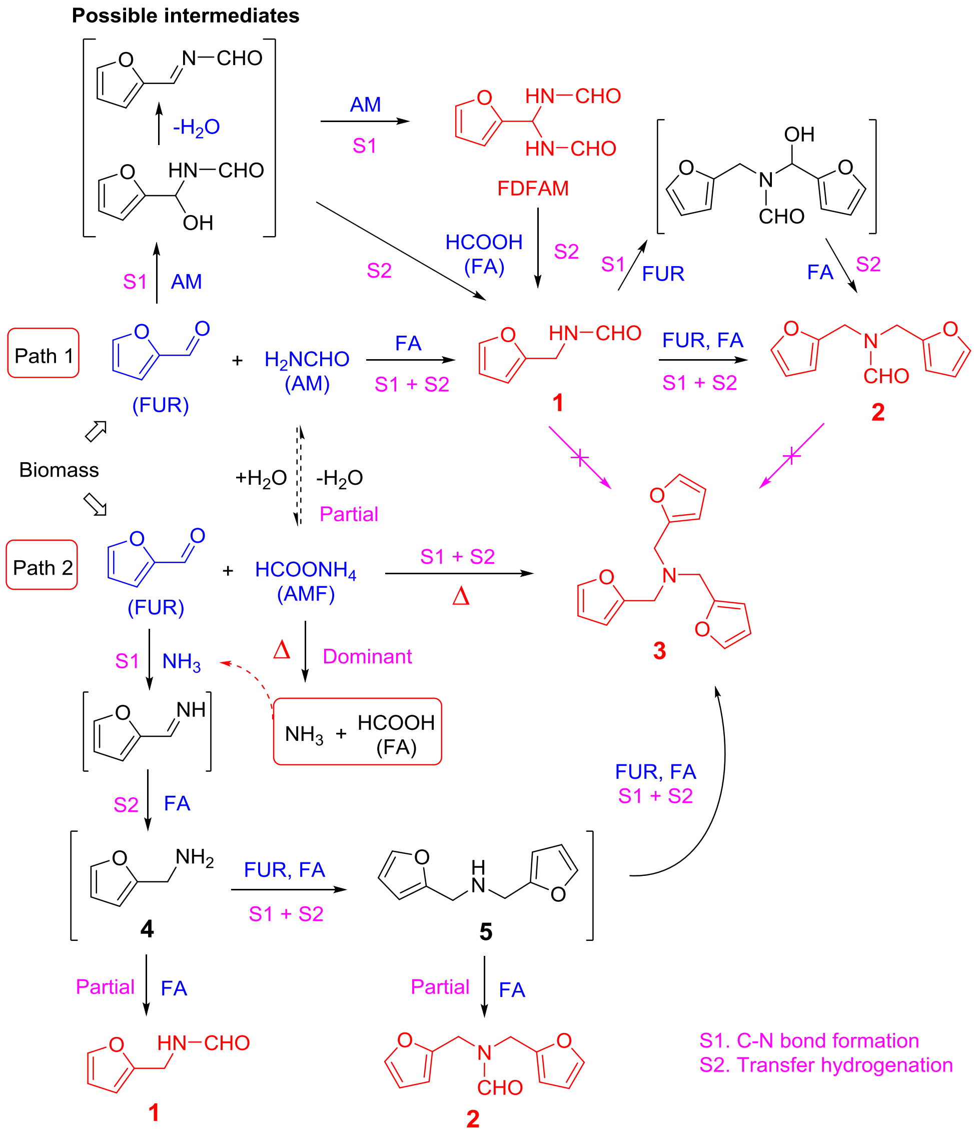

Supplementary Figure 25 Schematic illustration of reaction pathways for furfural (FUR) amination with formamide/formic acid (AM/FA) or ammonium formate (AMF).

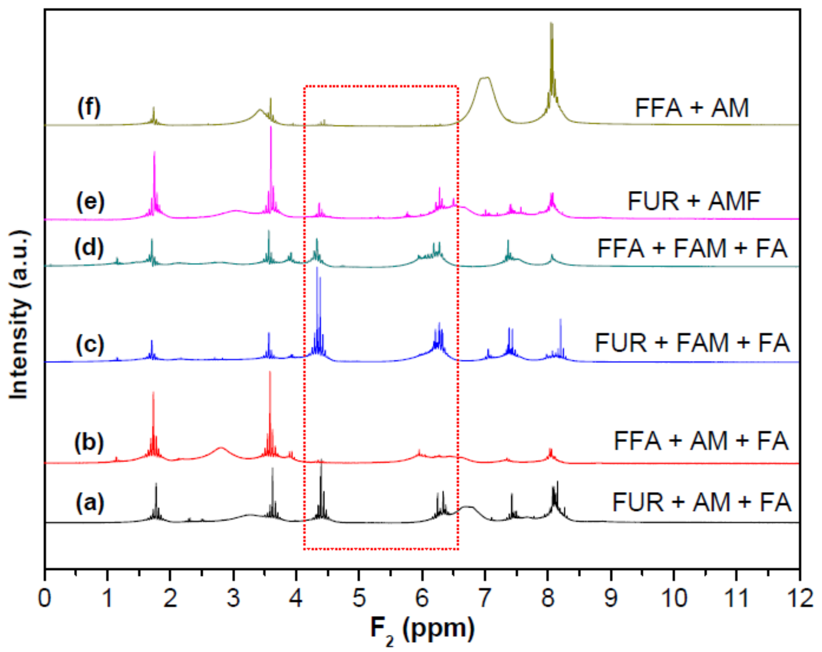

Supplementary Figure 26 ^1H NMR spectra of product solutions obtained from microwave irradiation of different starting materials that have been diluted with THF-d_8 (solvent peaks at 1.73 and 3.58 pm); reaction conditions: (a) 2 mmol FUR, 12 mmol AM, 6 mmol FA, 180 ºC, 3 min; (b) 2 mmol FFA, 12 mmol AM, 6 mmol FA, 180 ºC, 3 min; (c) 2 mmol FUR, 2 mmol FAM (**4**), 6 mmol FA, 180 ºC, 3 min; (d) 2 mmol FFA, 10 mmol FAM (**4**), 6 mmol FA, 180 ºC, 3 min; (e) 2 mmol FUR, 12 mmol AMF, 195 ºC, 5 min; (f) 2 mmol FFA, 12 mmol AM, 180 ºC, 10 min.

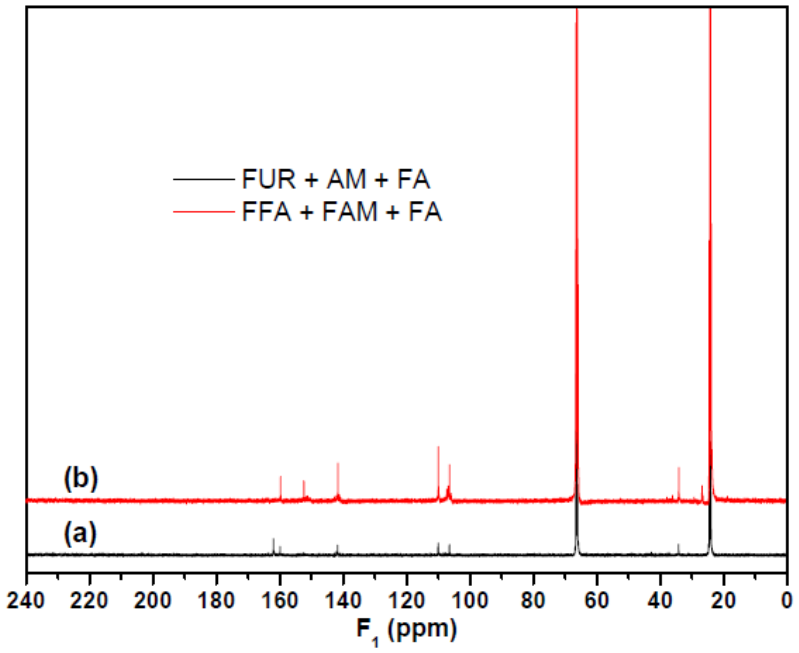

Supplementary Figure 27 ^{13}C NMR spectra of the reaction mixture diluted by THF-d_8 under microwave irradiation conditions: (a) 2 mmol FUR, 12 mmol AM, 6 mmol FA, 180 ºC, 3 min; (b) 2 mmol FFA, 10 mmol FAM (**4**), 6 mmol FA, 180 ºC, 3 min.

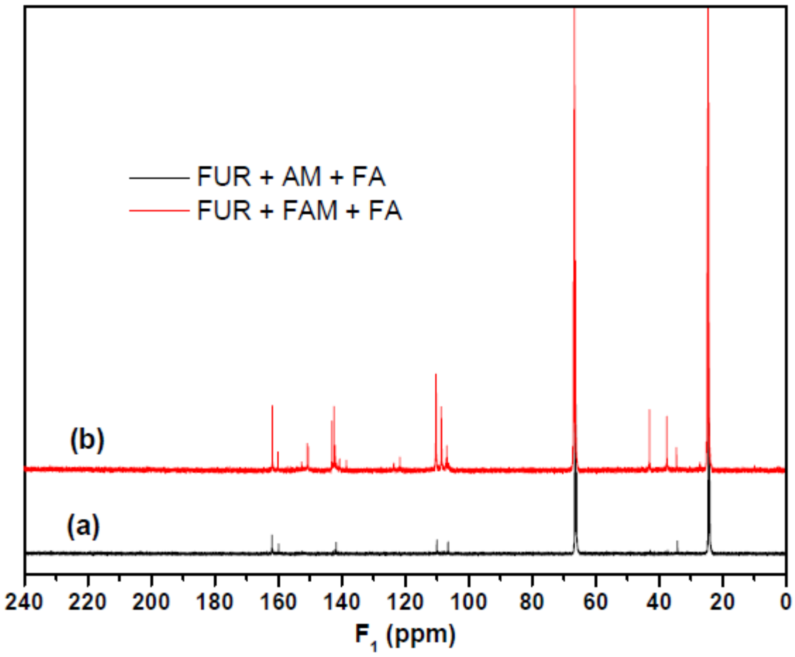

Supplementary Figure 28 ^{13}C NMR spectra of product solutions obtained under microwave irradiation that have been diluted by THF-d_8.

Conditions: (a) 2 mmol FUR, 12 mmol AM, 6 mmol FA, 180 ºC, 3 min; (b) 2 mmol FUR, 2 mmol FAM (**4**), 6 mmol FA, 180 ºC, 3 min.

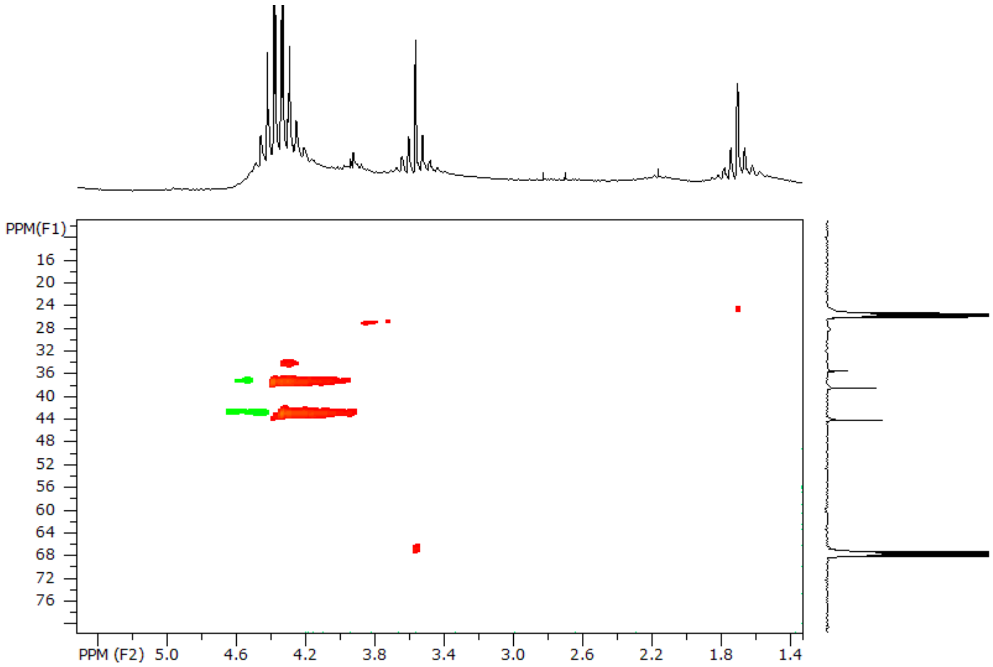

Supplementary Figure 29 ^{1}H-^{13}C HSQC spectra of product solution obtained from microwave irradiation that has been diluted by THF-d_8.

Conditions: 2 mmol FUR, 2 mmol FAM (**4**), 6 mmol FA, 180 ºC, 3 min.

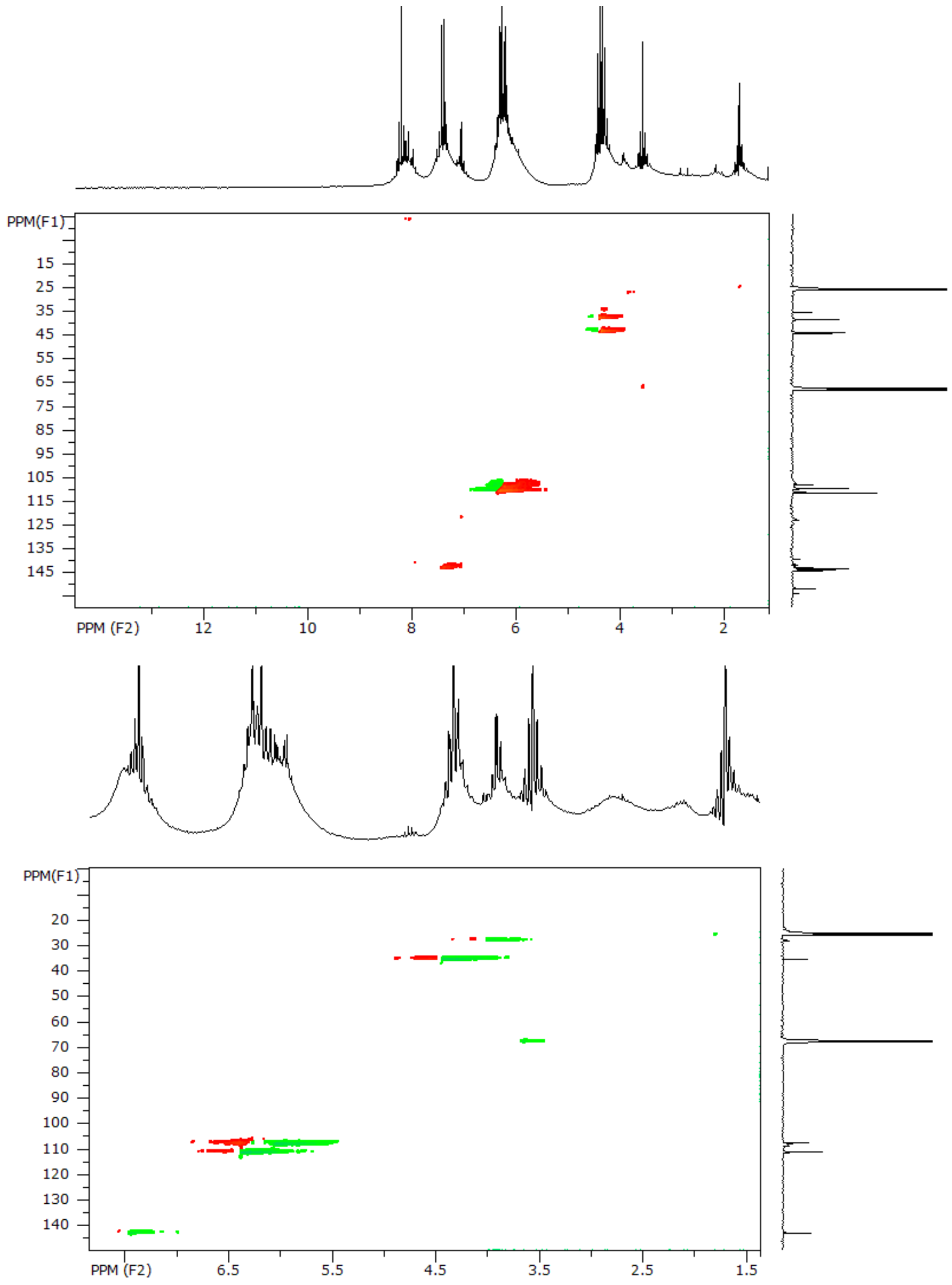

Supplementary Figure 30 ^{1}H-^{13}C HSQC spectra of product solutions obtained by microwave irradiation that have been diluted by THF-d_8. Conditions: (top) 2 mmol FUR, 2 mmol FAM (**4**), 6 mmol FA, 180 ºC, 3 min; (bottom) 2 mmol FFA, 10 mmol FAM (**4**), 6 mmol FA, 180 ºC, 5 min.

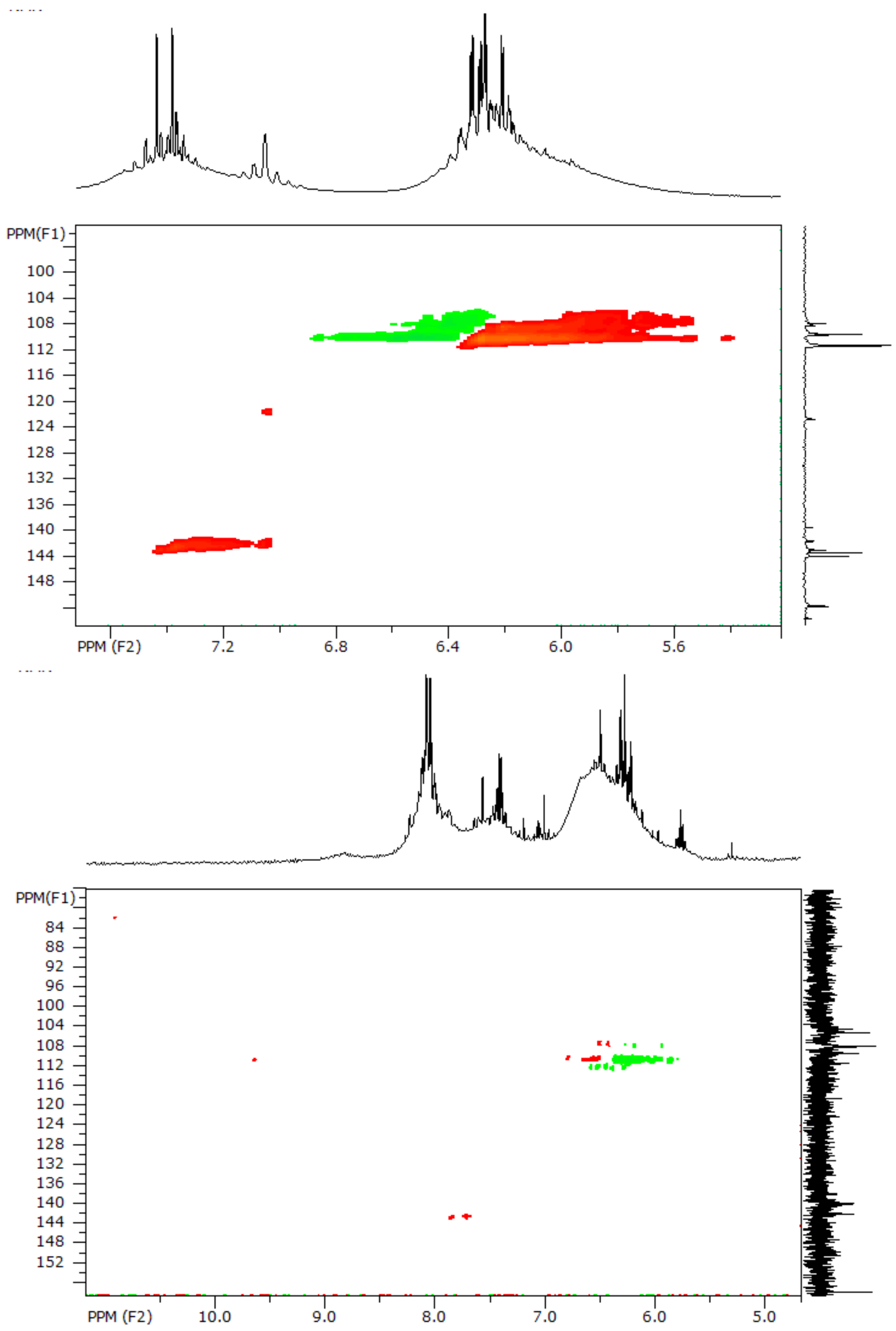

Supplementary Figure 31 ^{1}H-^{13}C HSQC spectra of product solutions obtained from microwave irradiation that have been diluted by THF-d_8. Conditions: (top) 2 mmol FUR, 2 mmol FAM (**4**), 6 mmol FA, 180 ºC, 3 min; (bottom) 2 mmol FUR, 12 mmol AMF, 195 ºC, 5 min.

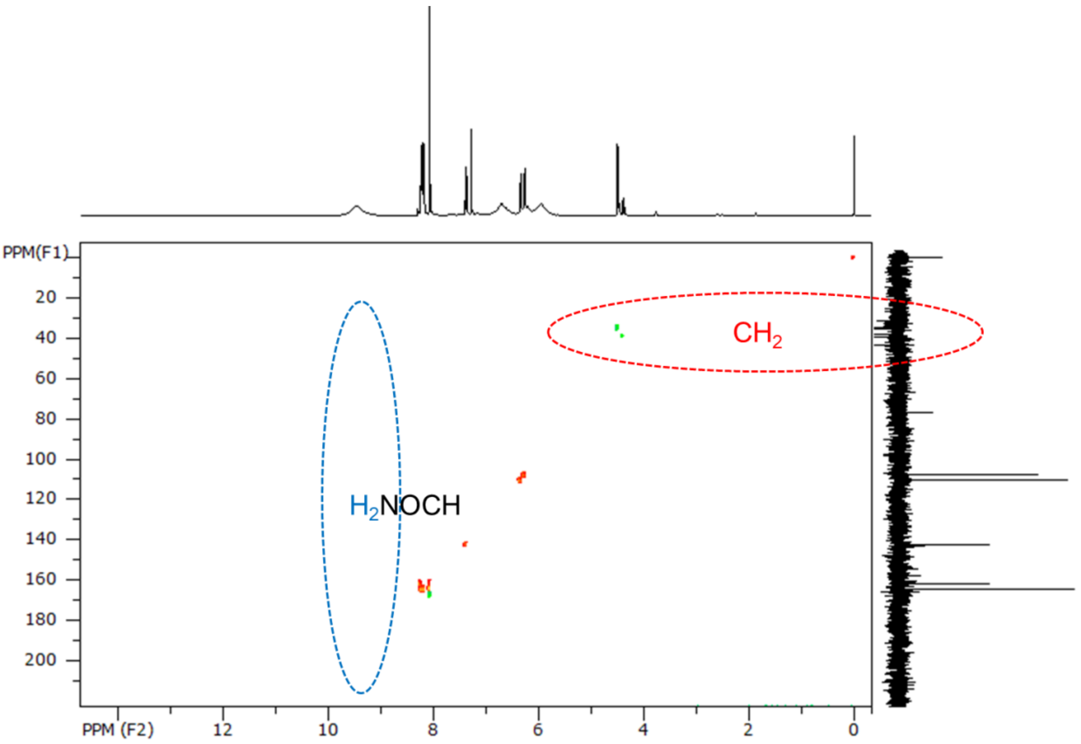

Supplementary Figure 32 ^{1}H-^{13}C NMR HSQC-DEPT spectrum of microwave-assisted amination of furfural (FUR) and formamide (AM) using deuterium-labeled formic acid (FA).

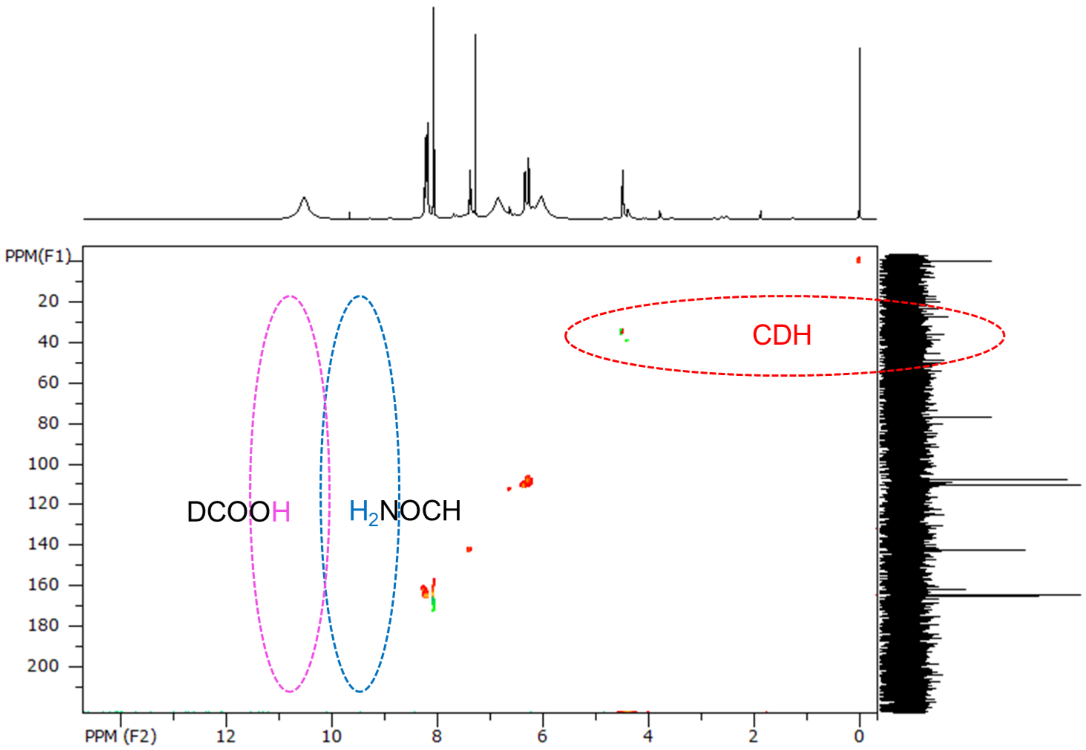

Supplementary Figure 33 ^{1}H-^{13}C NMR HSQC-DEPT spectrum of microwave-assisted amination of furfural (FUR) and formamide (AM) using deuterium-labeled formic acid (FA).

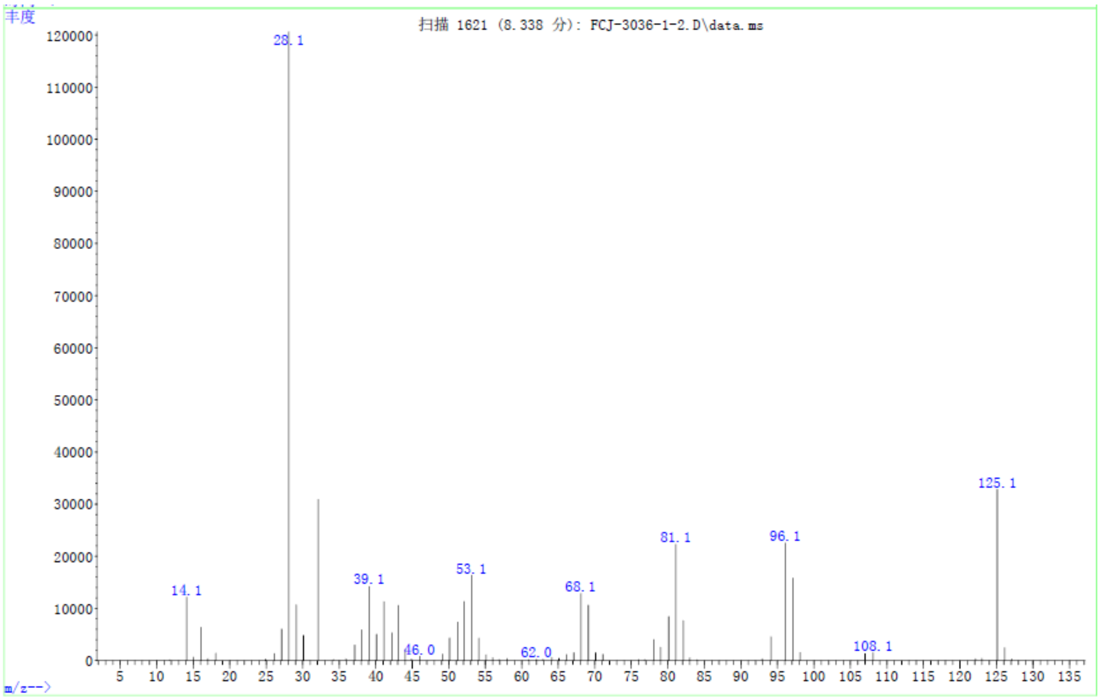

Supplementary Figure 34 Mass spectrum of microwave-assisted amination of furfural with formamide using undeuterated formic acid, abundance versus m/z, scan (1621), 8.338 min.

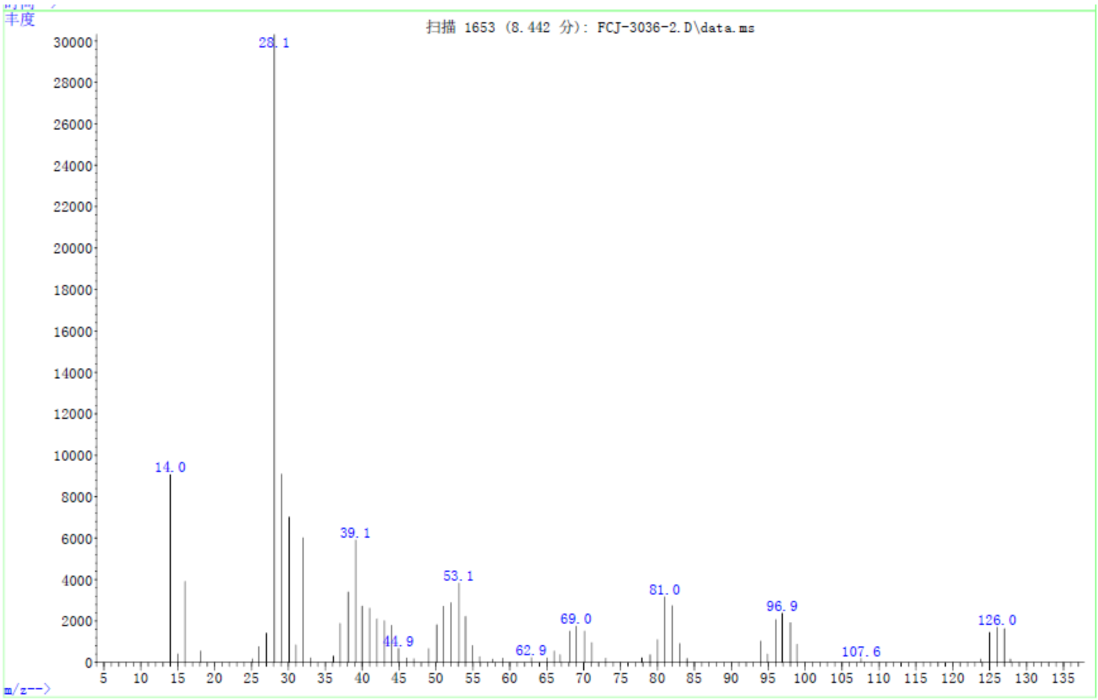

Supplementary Figure 35 Mass spectrum of microwave-assisted amination of furfural with formamide using deuterium-labeled formic acid (FA); abundance versus m/z, scan (1653), 8.442 min.

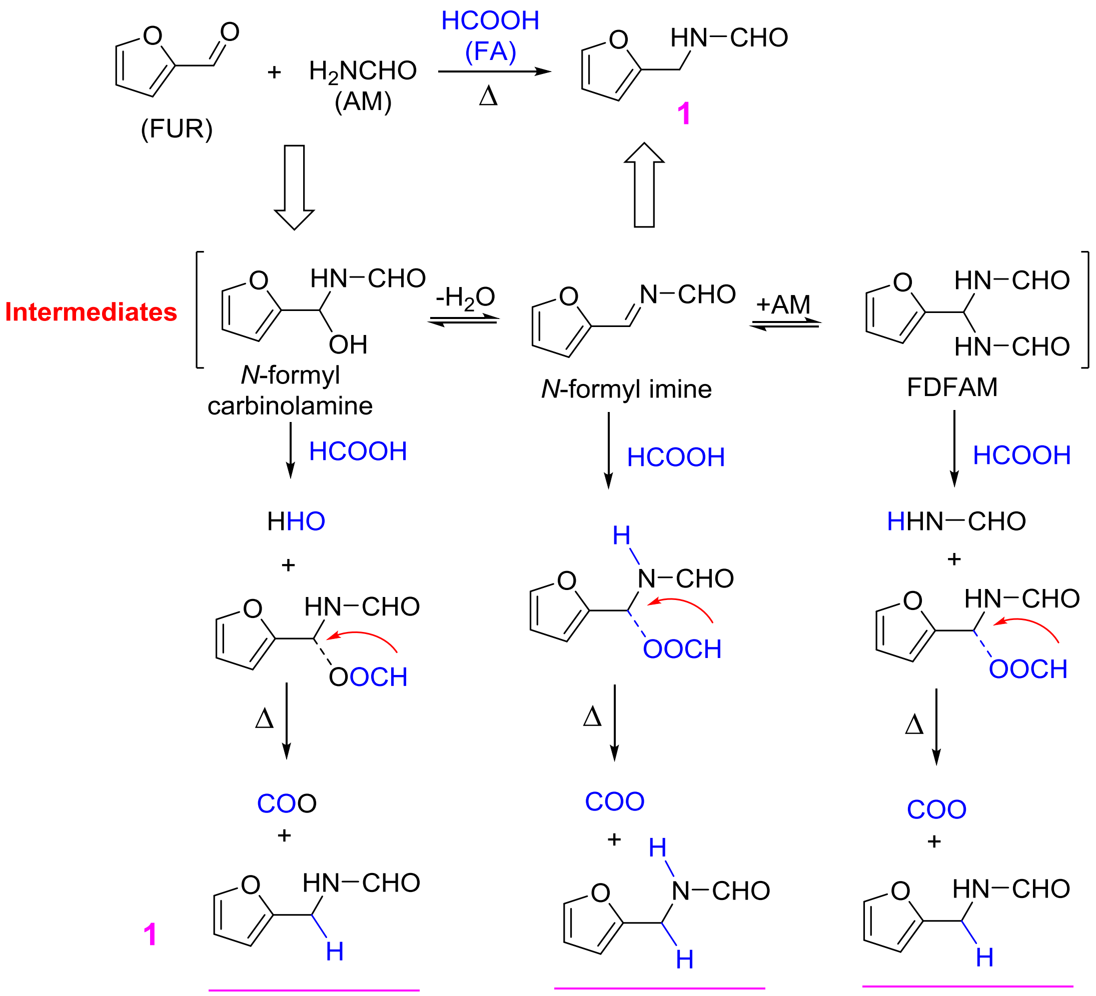

Supplementary Figure 36 Schematic illustration of transfer hydrogenation process for the synthesis of *N*-(furan-2-ylmethyl)formamide (**1**) from amination of furfural with formamide and formic acid via several intermediates.

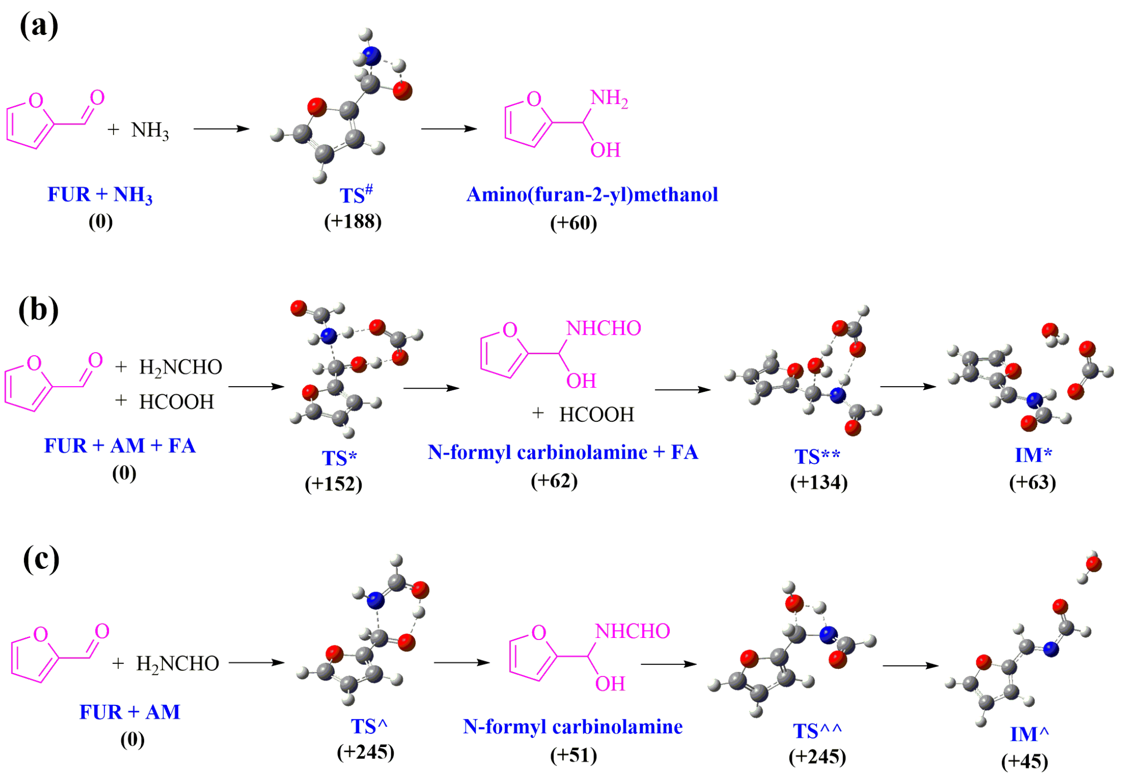

Supplementary Figure 37 Computed free energy profiles for the amination of furfural (FUR) with NH_3 **(a)** or formamide (AM) with **(b)** or without **(c)** formic acid (FA) in the absence of metal catalyst. TS: transition state, IM: intermediate. Values in parentheses are free energies (kJ mol^{-1}) with respect to the total energy of the separated reactant molecules.

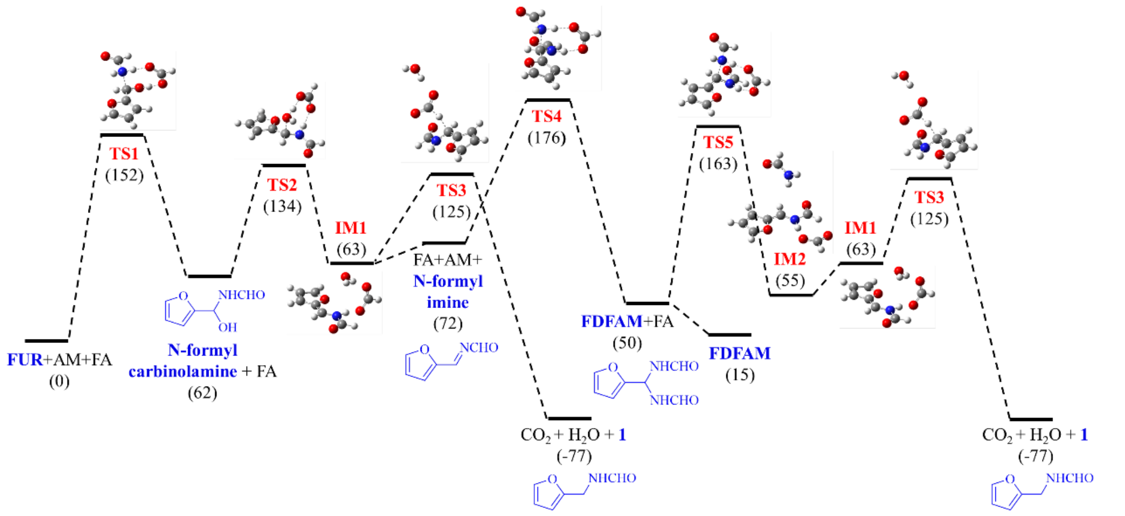

Supplementary Figure 38 Computed free energy profiles in a different format (not to scale) to Fig. 4 [T = 180 ºC, B3LYP/6-311+G(2s,2p)] for the amination of FUR and AM using FA to the target product **1** via N-formyl carbinolamine, N-formyl imine, and FDFAM. TS: transition state, IM: intermediate. Values in parentheses are free energies (kJ mol^{-1}) with respect to the starting energy of the three separated reactants FUR, AM and FA.

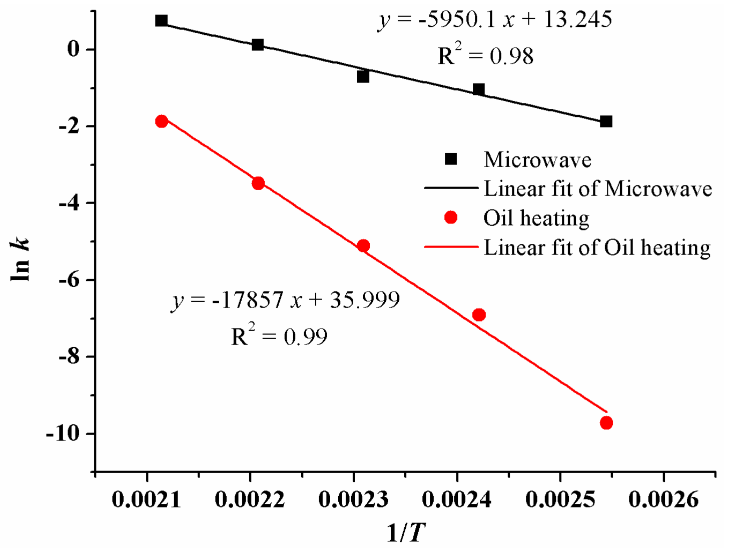

Supplementary Figure 39 Arrhenius plots for the amination of FUR and AM using FA under microwave (Ea =49.5 kJ mol^{-1}) and oil-heating conditions (Ea= 148 kJ mol^{-1}).

Assuming that the amination of FUR and AM with FA is a 1st order process, the reaction rate is expressed as: k[FUR] = d[FUR]/dt. After integration, the relationship becomes the well-known form: -ln(1 - X) = kt + C with X being the conversion of FUR. Values of -ln(1 - X) were plotted against reaction time (t) at different temperatures to obtain rate constants (k), as shown in Supplementary Figure 39. The activation energy (Ea) was calculated from rate constants by the Arrhenius equation (ln$k$ = -Ea/RT + lnA).

Ea (oil) = 17857 × 8.314 J mol^{-1} = 148 kJ mol^{-1}

Ea (microwave) = 5950.1 × 8.314 J mol^{-1} = 49.5 kJ mol^{-1}

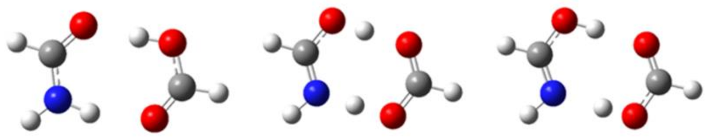

IS
0

TS
G = 41.5 kJ mol^{-1}
HF = 50.3 kJ mol^{-1}

FS
G = 43.5 kJ mol^{-1}
HF = 41.5 kJ mol^{-1}

Supplementary Figure 40 Most probable activation/interaction model between formamide (AM) and formic acid (FA) with calculated free energies. IS: initial state, TS: transition state, FS: final state.

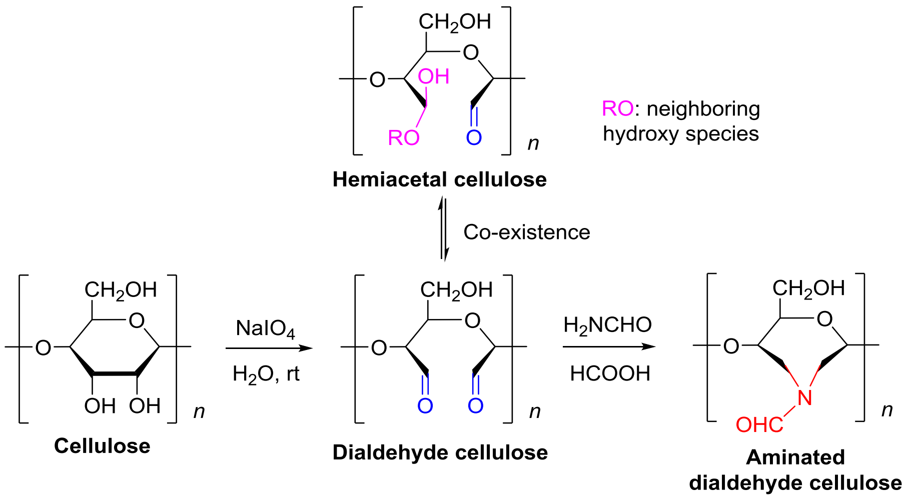

Supplementary Figure 41 Schematic illustration of upgrading cellulose to the *N*-containing products via dialdehyde cellulose.

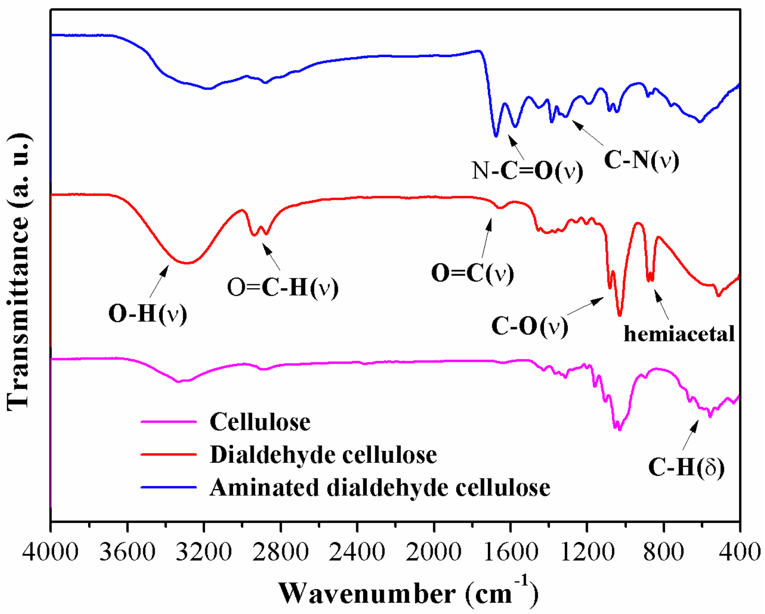

Supplementary Figure 42 FT-IR spectra of cellulose, dialdehyde cellulose, and aminated dialdehyde cellulose.

During the pre-oxidation of cellulose using $NaIO_4$, the formed dialdehydes cellulose was partially converted into the hemiacetalized cellulose (co-existent) by reacting with the neighboring hydroxy groups (Supplementary Figure 41) [2, 3]. Both of them were found to be capable of undergoing amination to give the *N*-formyl-containing product, as illustrated by FT-IR (Supplementary Figure 42). The aminated product was estimated to be above 85%, based on the nitrogen content (elemental analysis) relative to the theoretically formed aldehyde.

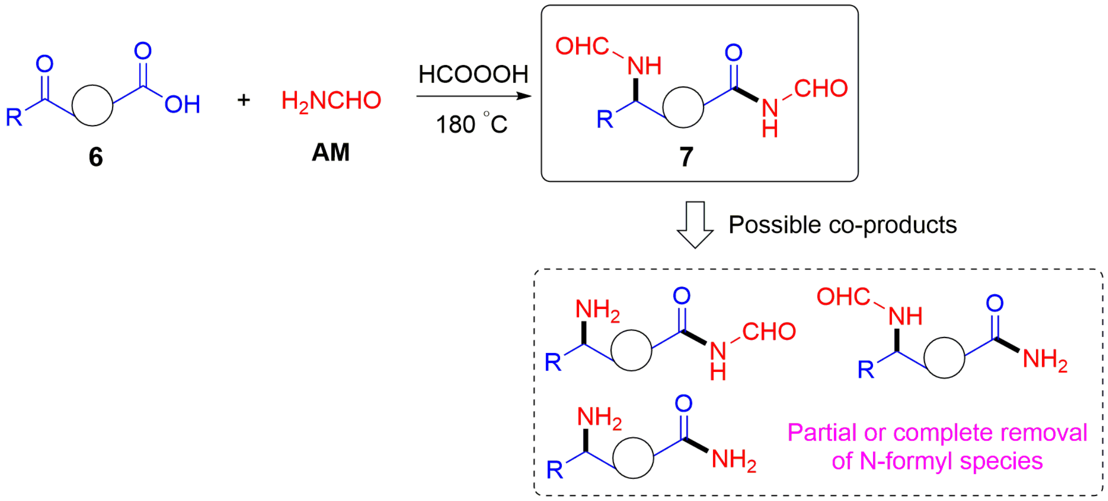

Supplementary Figure 43 Possible co-products formed by partial or complete removal of N-formyl species.

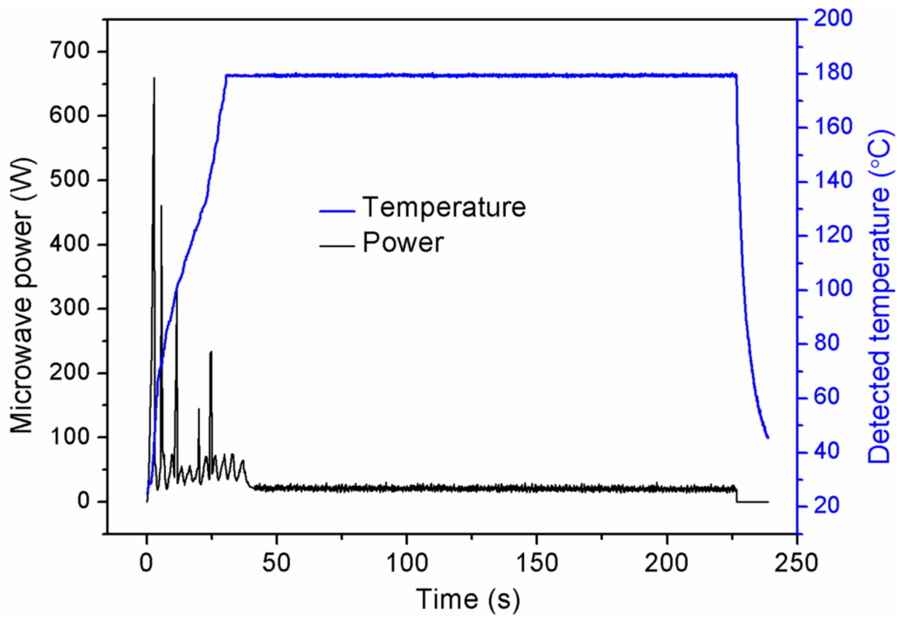

Supplementary Figure 44 Representative profiles of temperature and power plotted with time for the microwave reactor conducted at 180 ºC (heating time: ca. 30 s, and cooling time: ca. 20 s).

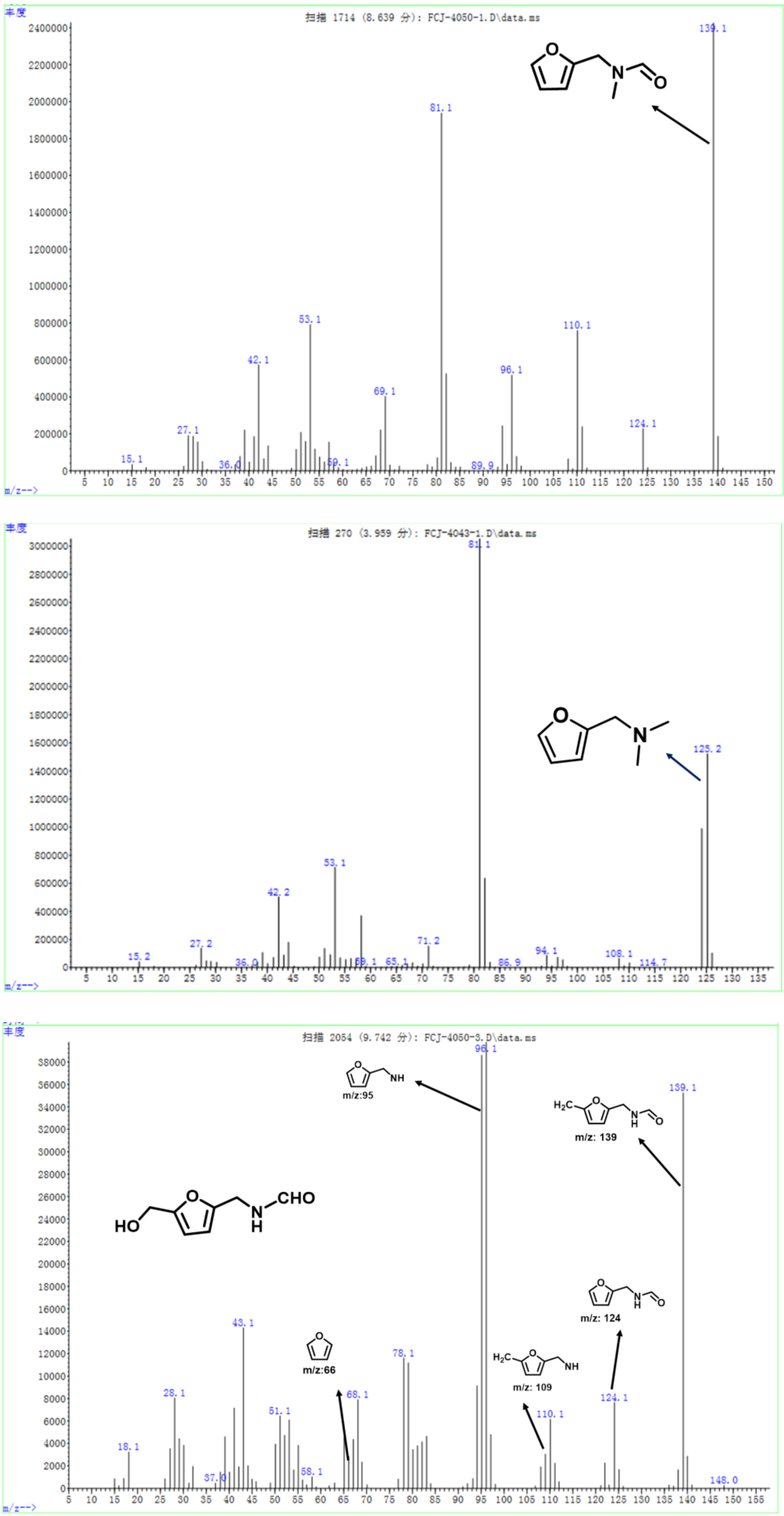

Supplementary Figure 45 GC-MS spectra of representative products from furanic aldehydes

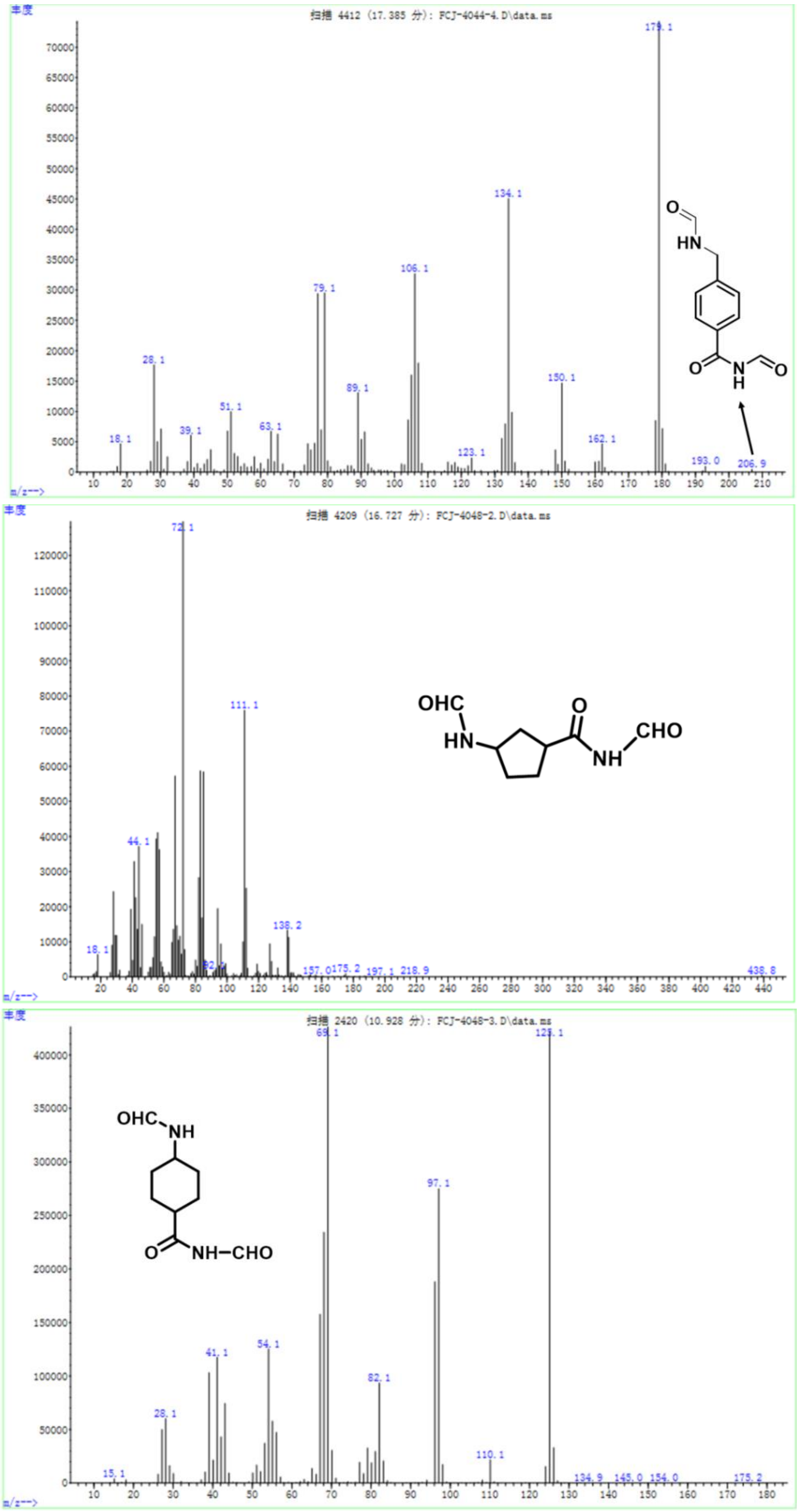

Supplementary Figure 46 GC-MS spectra of representative products from oxocarboxylic acids

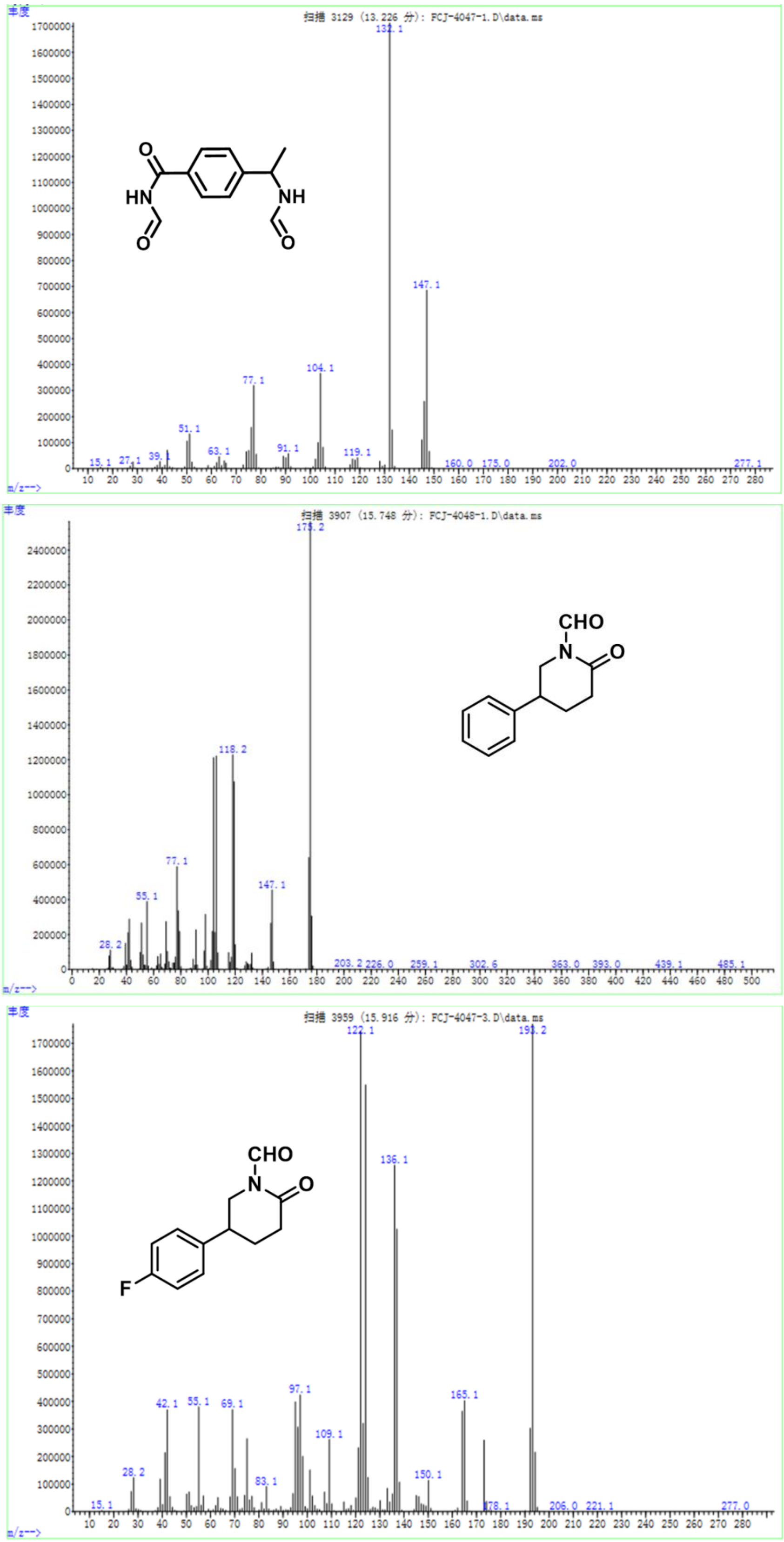

Supplementary Figure 47 GC-MS spectra of representative products from keto acids

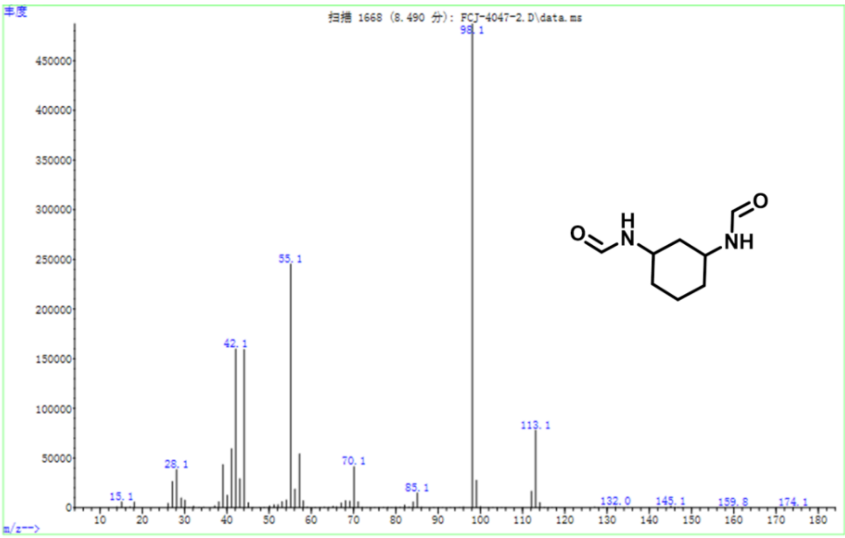

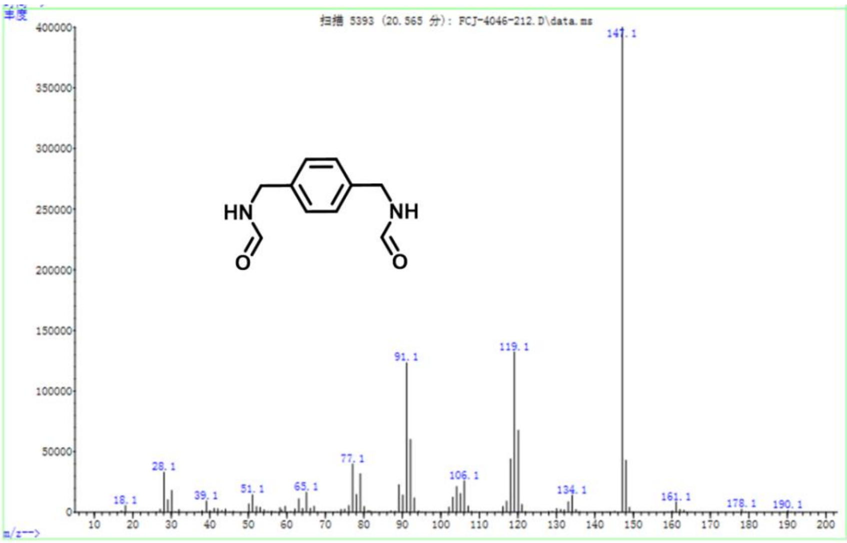

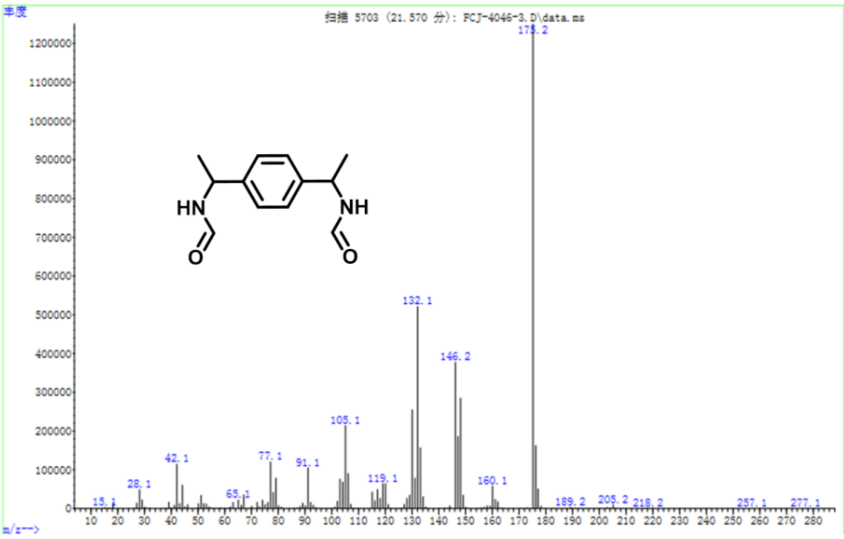

Supplementary Figure 48 GC-MS spectra of representative products from dicarbonyl compounds

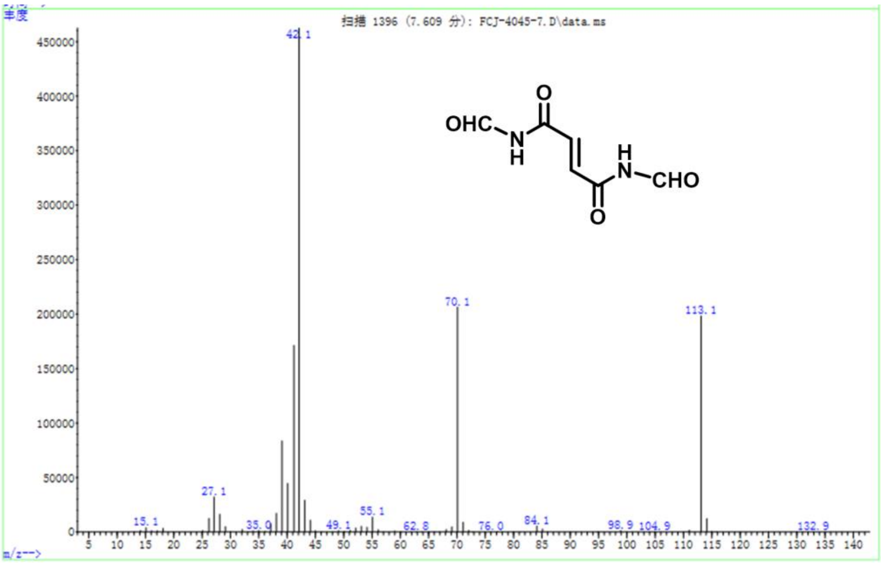

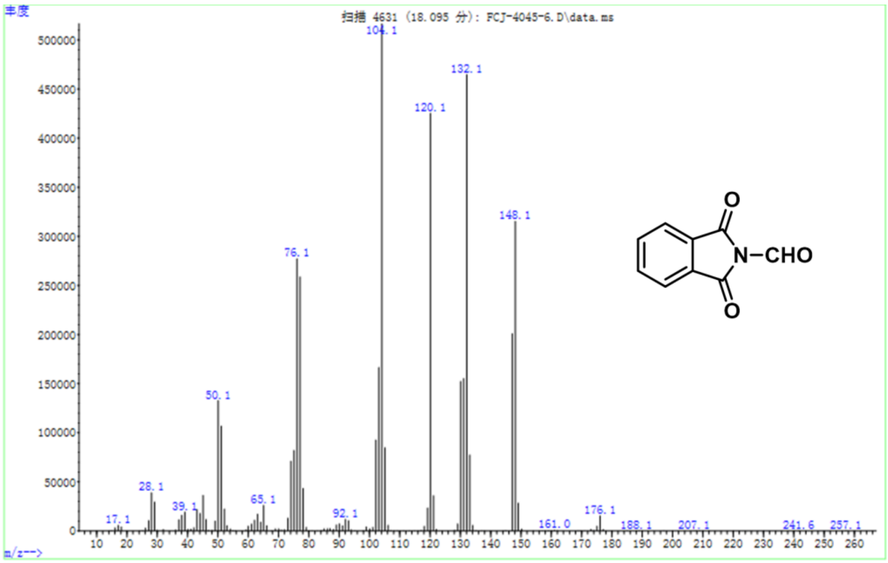

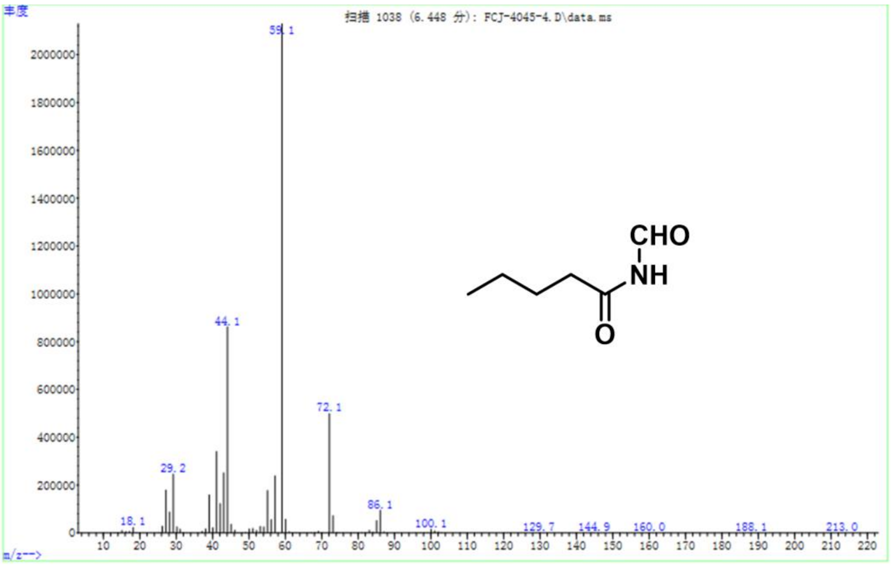

Supplementary Figure 49 GC-MS spectra of representative products from carboxylic acids

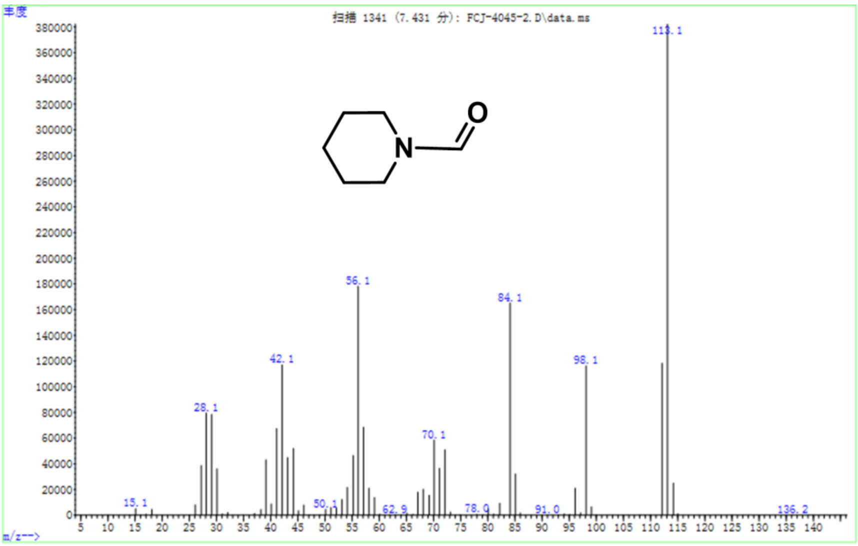

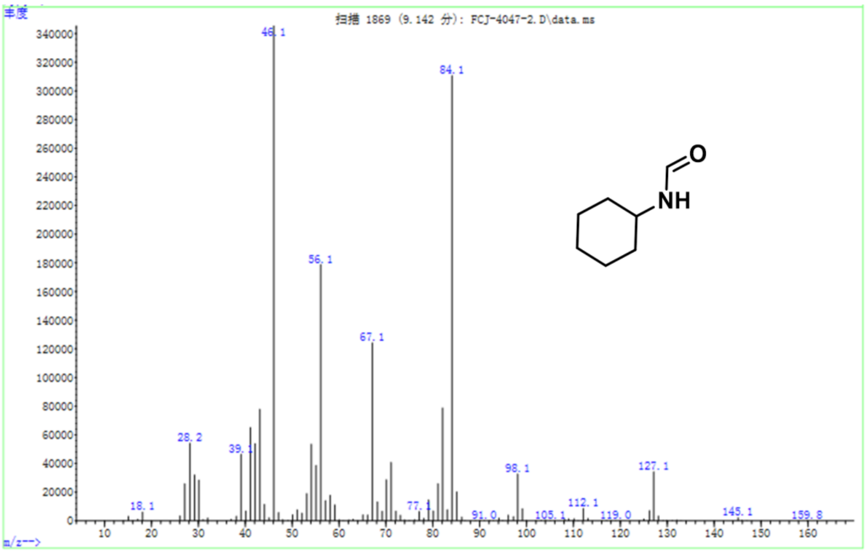

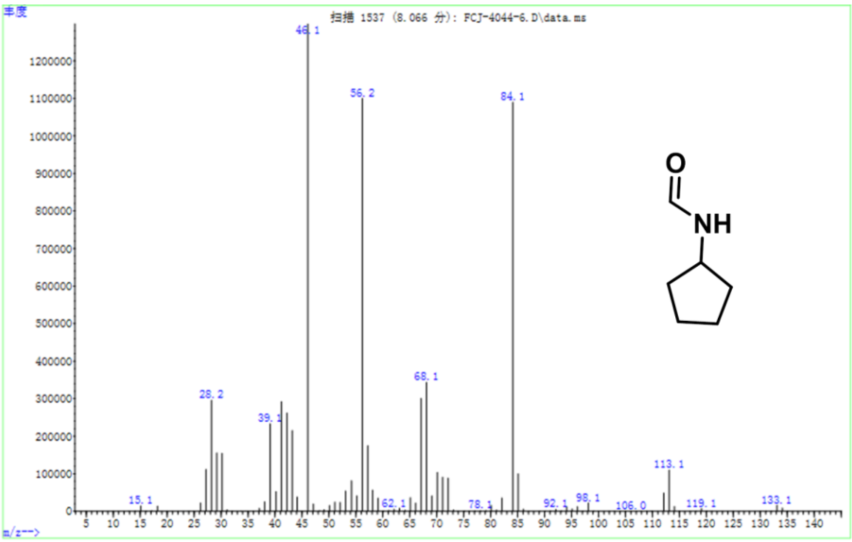

Supplementary Figure 50 GC-MS spectra of representative products from carboxides

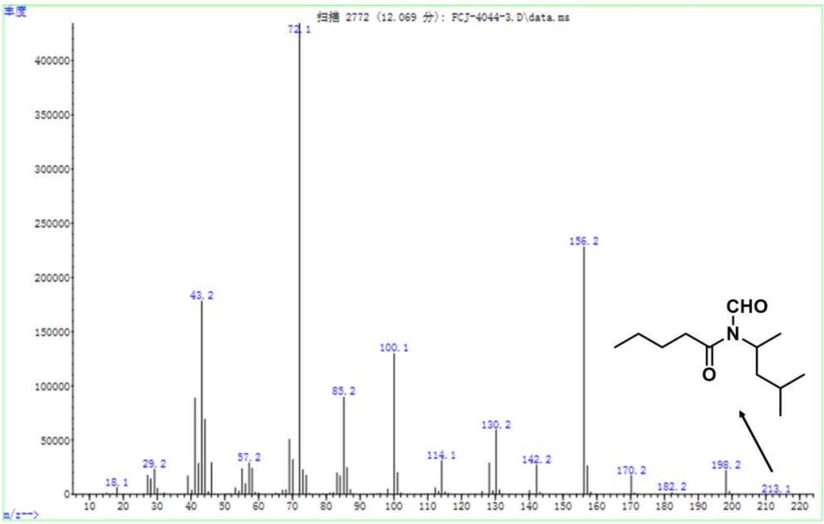

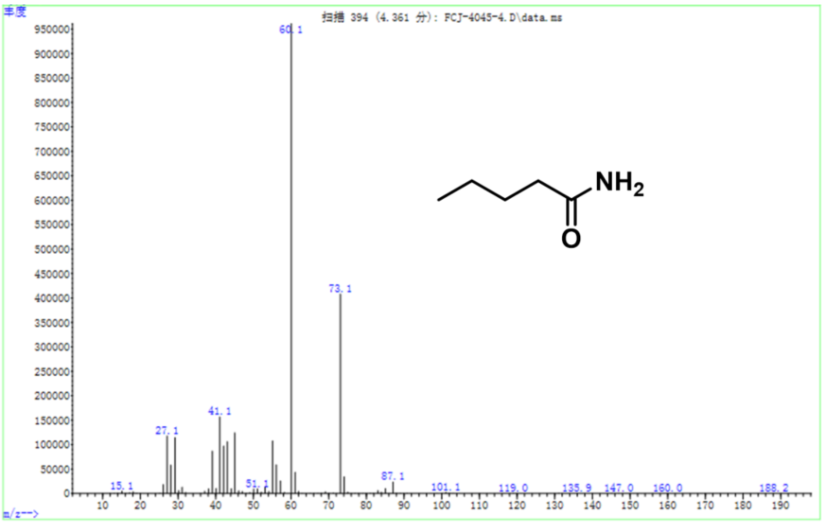

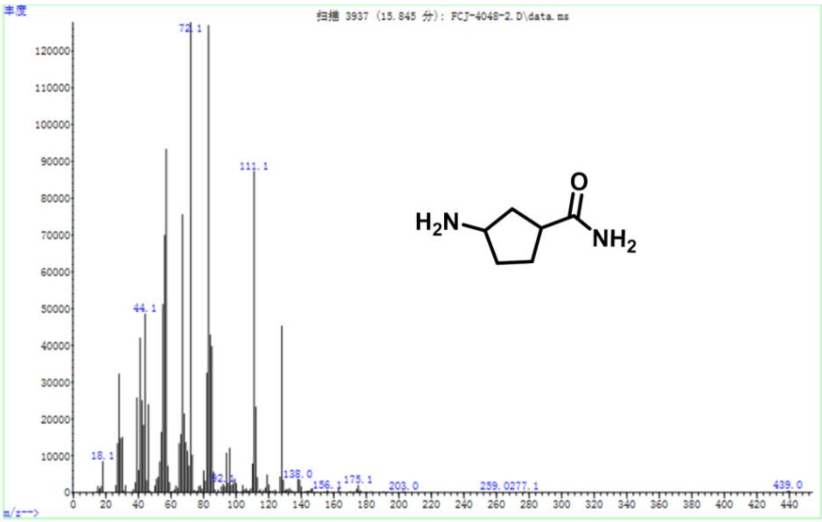

Supplementary Figure 51 GC-MS spectra of representative products from ketones and/or acids

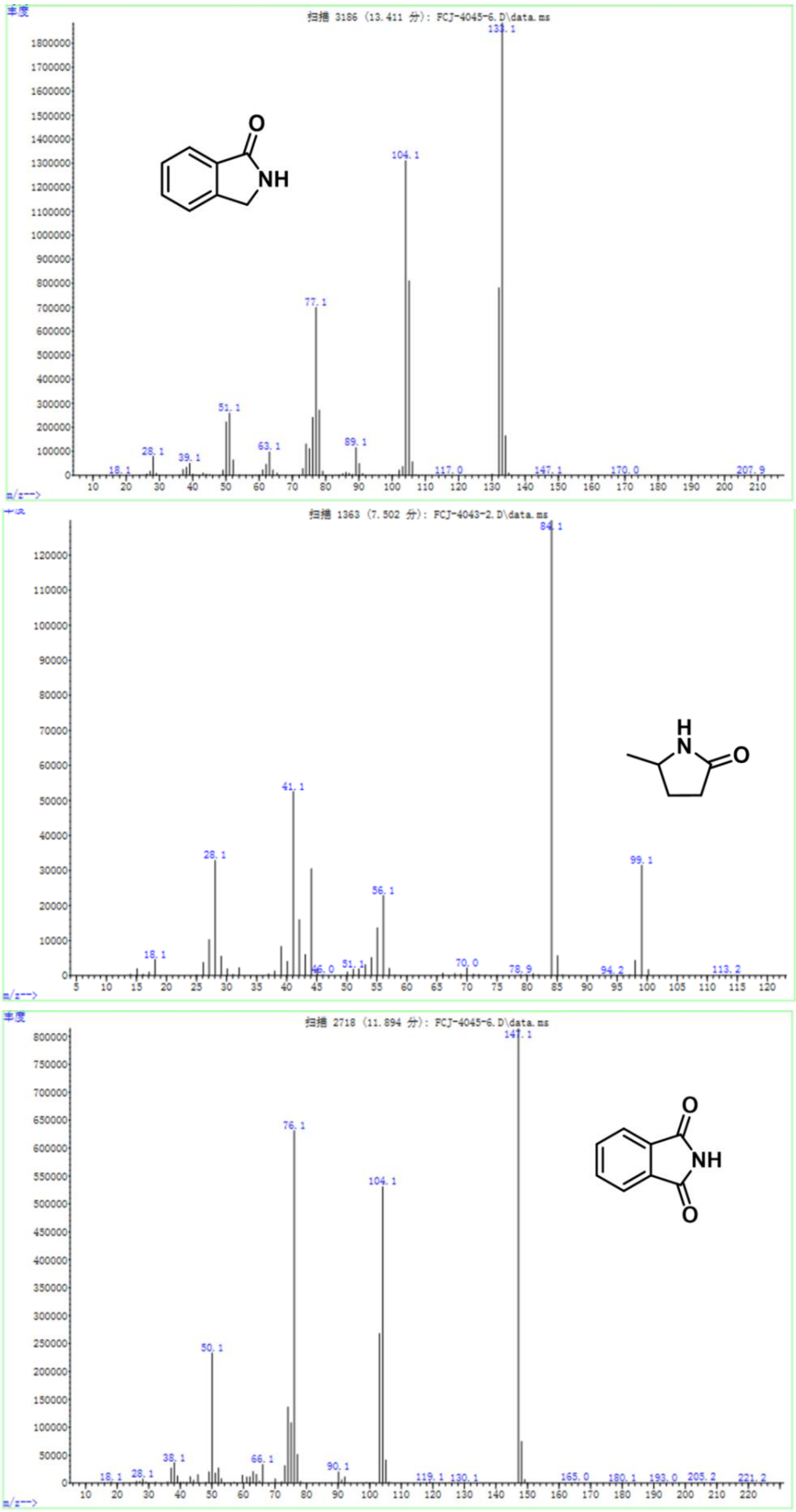

Supplementary Figure 52 GC-MS spectra of representative products without N-formyl group

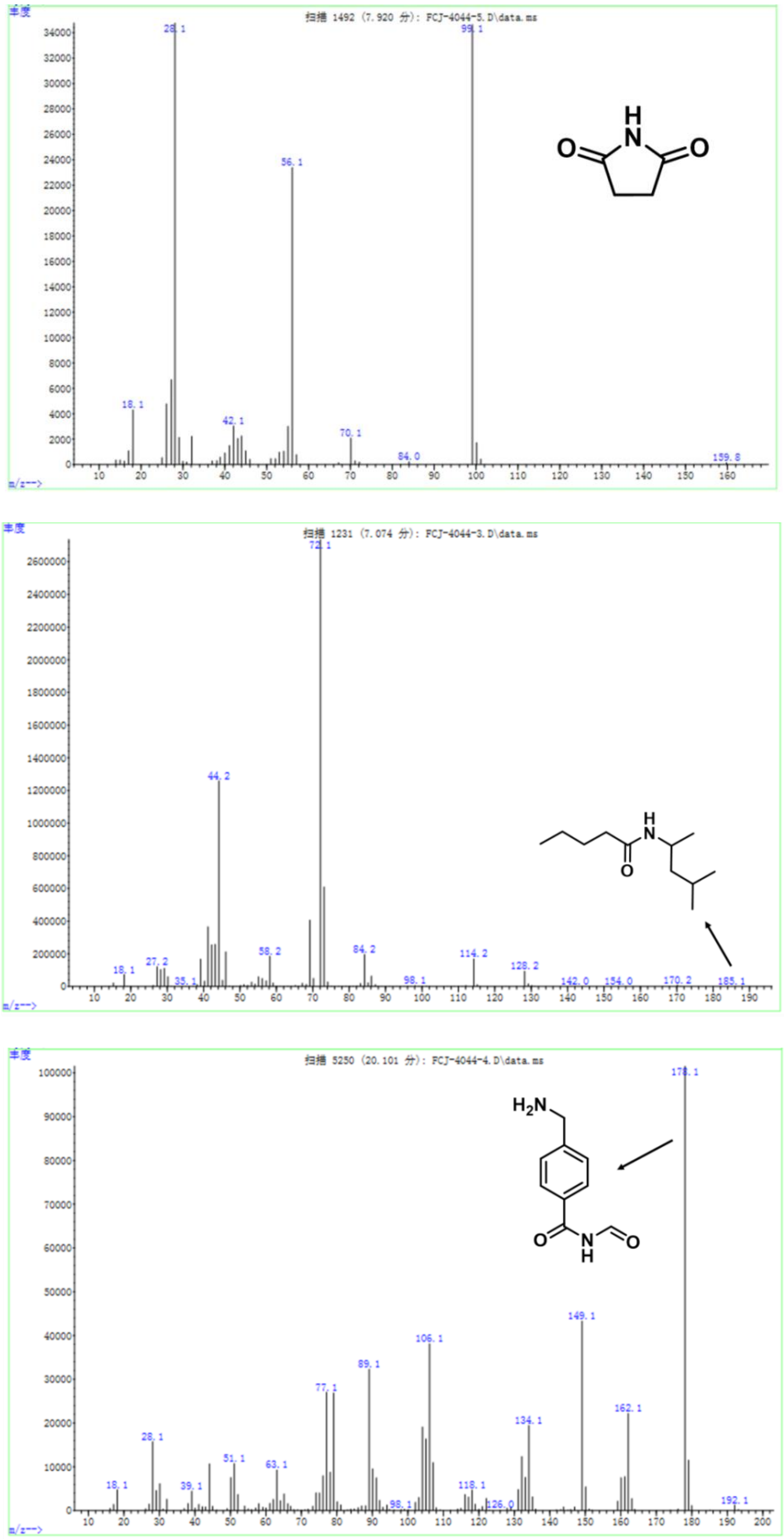

Supplementary Figure 53 GC-MS spectra of representative products obtained via intra- and inter-condensation or without condensation reaction

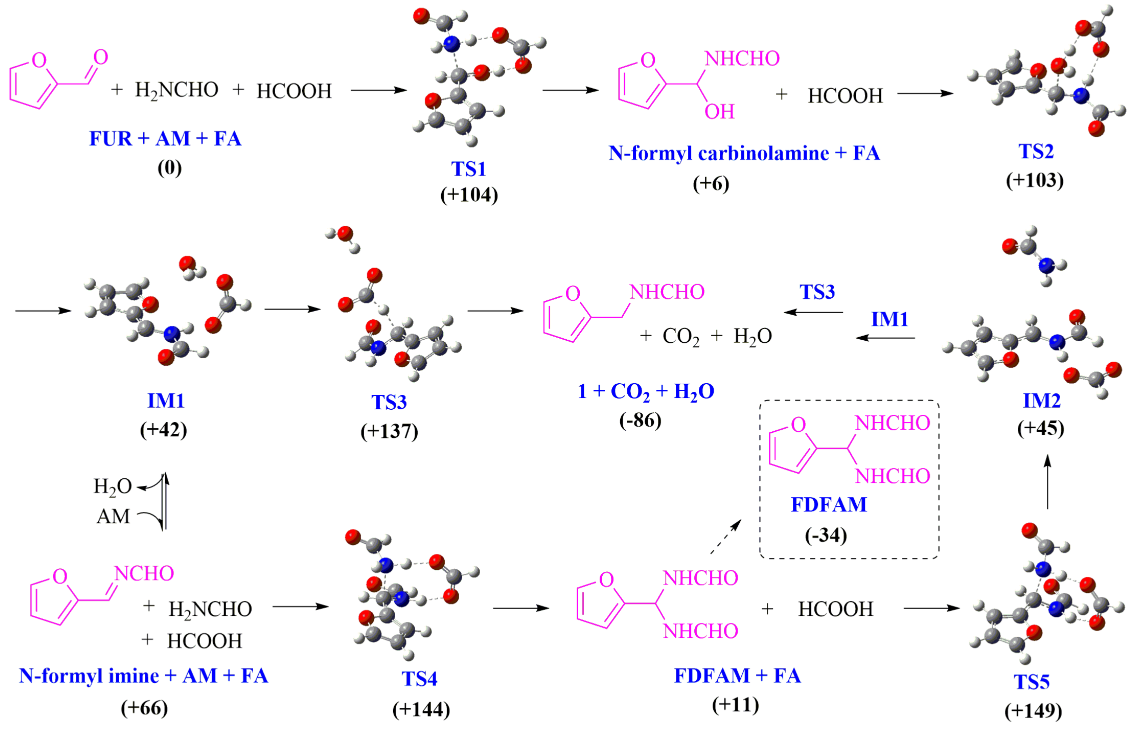

Supplementary Figure 54 Computed free energy profiles [T = 180 ºC, M06-2X/def2-TZVP] for the amination of FUR and AM using FA to the target product **1** via *N*-formyl carbinolamine, *N*-formyl imine, and FDFAM. TS: transition state, IM: intermediate. Values in parentheses are free energies (kJ mol^{-1}) with respect to the starting energy of the three separated reactants FUR, AM and FA.

Panel A: 3D figures for **1** synthesis via N-formyl carbinolamine

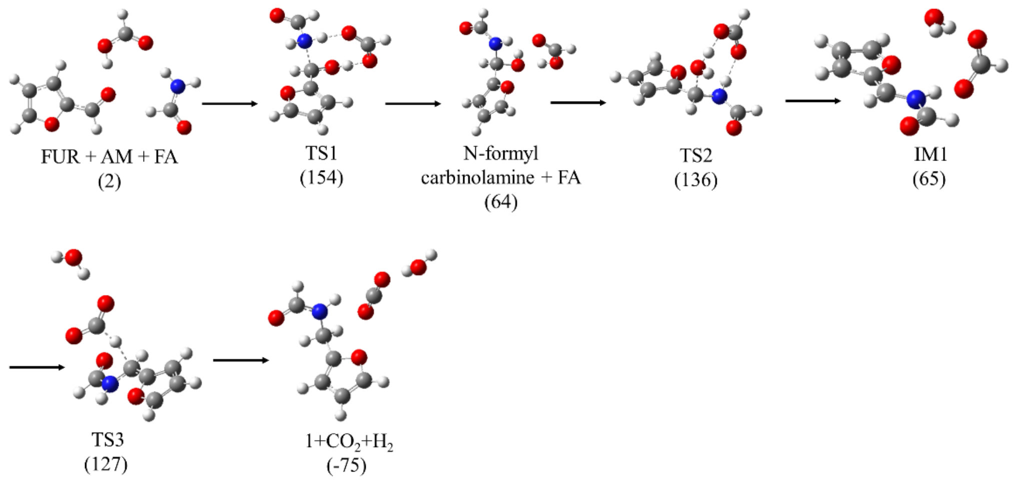

Panel B: 3D figures for **1** synthesis via N-Formyl imine and FDFAM without FA in the second step

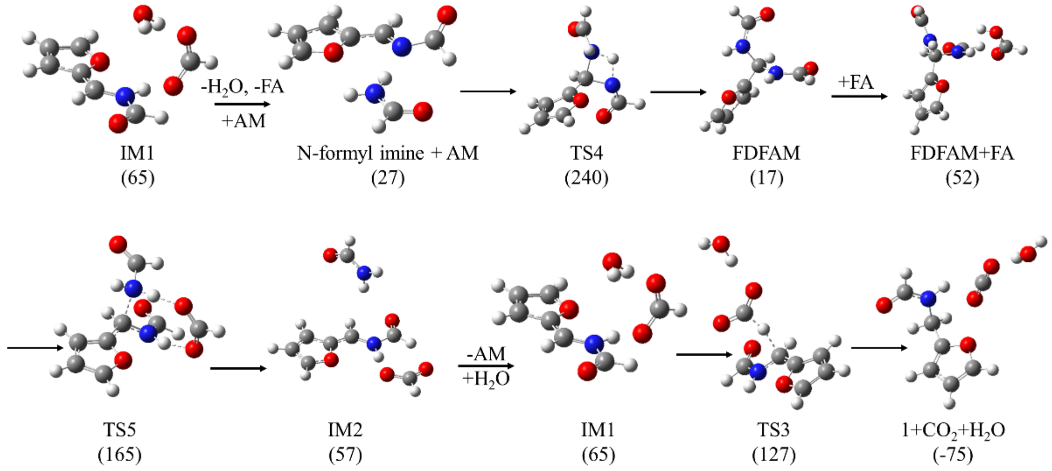

Panel C: 3D figures for **1** synthesis via N-Formyl imine and FDFAM with FA in the second step

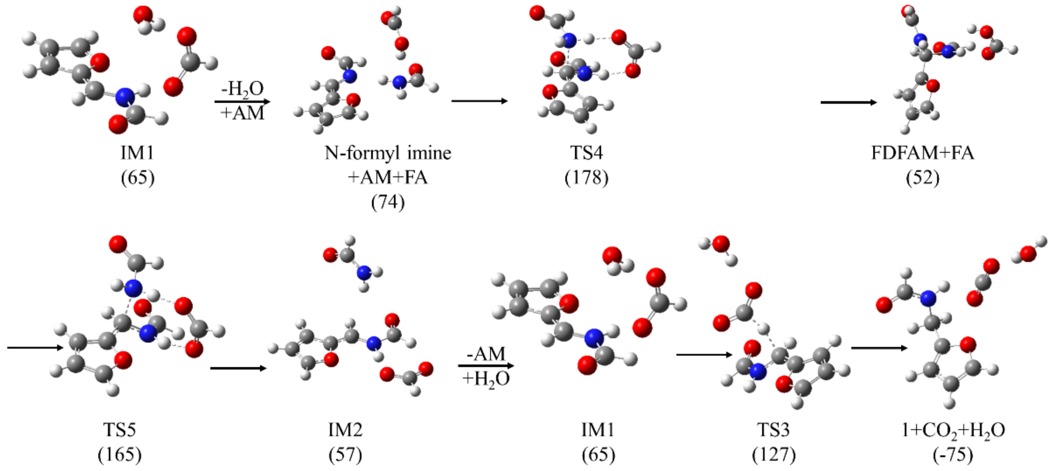

Supplementary Figure 55 Structures with free energies for the reaction systems via N-formyl carbinolamine, or N-Formyl imine and FDFAM, calculated on the basis of B3LYP/6-311+G(2s,2p) function at 180 °C. Values in parentheses are free energies (kJ mol^{-1}) with respect to the starting energy of the three separated reactants FUR, AM and FA.

Panel A: 3D figures for **1** synthesis via N-formyl carbinolamine

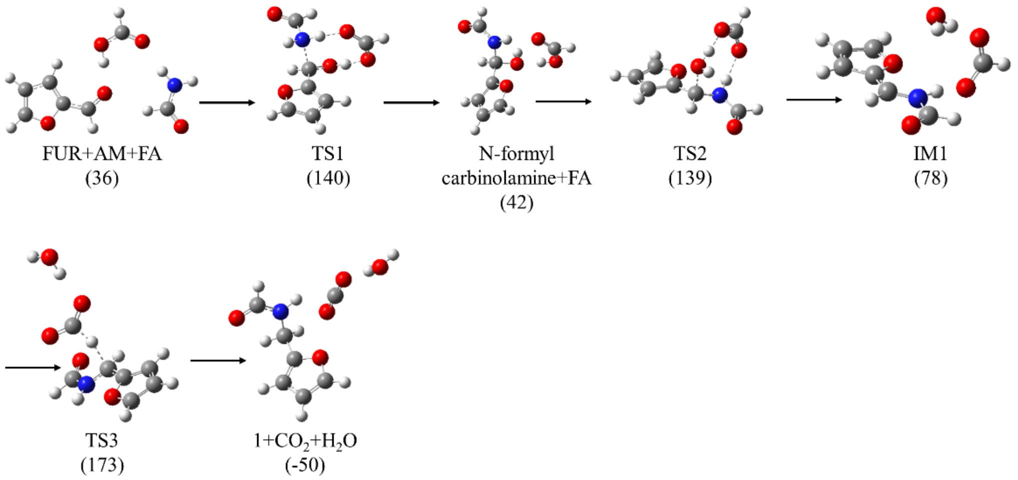

Panel B: 3D figures for **1** synthesis via N-Formyl imine and FDFAM without FA in the second step

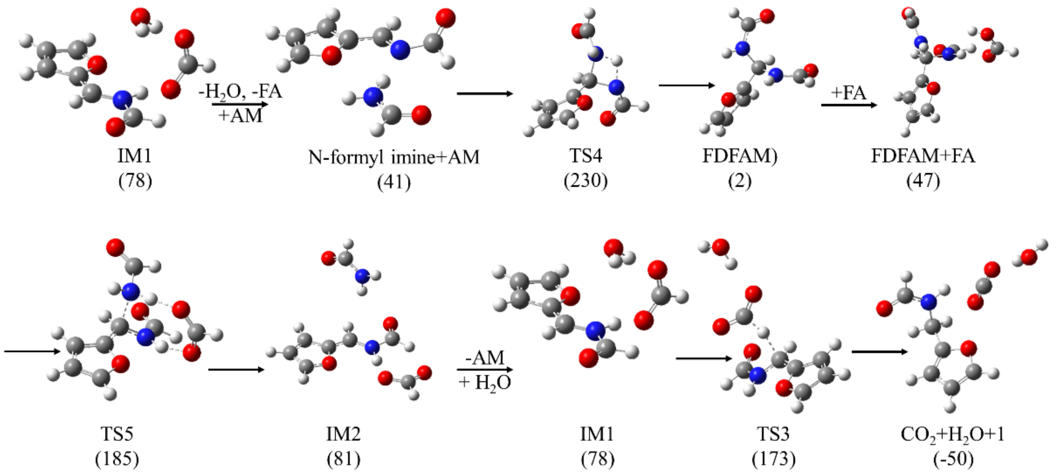

Panel C: 3D figures for **1** synthesis via N-Formyl imine and FDFAM with FA in the second step

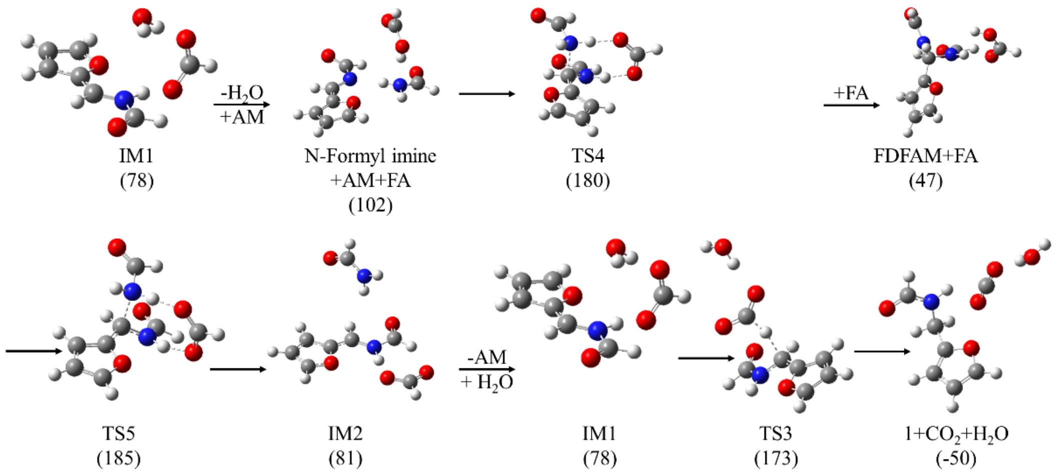

Supplementary Figure 56 Structures with free energies for the reaction systems via N-formyl carbinolamine, or N-Formyl imine and FDFAM, calculated on the basis of M06-2X/def2TZVP function at 180 °C. Values in parentheses are free energies (kJ mol^{-1}) with respect to the starting energy of the three separated reactants FUR, AM and FA.

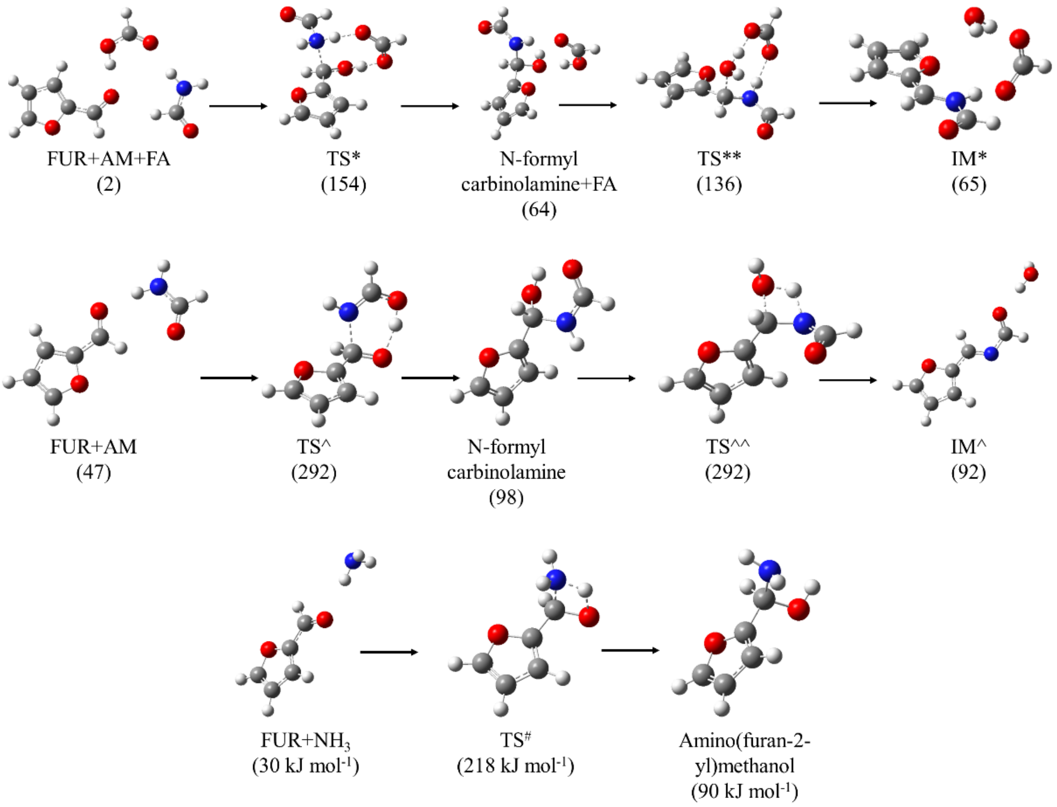

Supplementary Figure 57 Structures with free energies for the reaction systems (listed in Supplementary Figure 37), calculated on the basis of B3LYP/6-311+G(2s,2p) function at 180 °C. Values in parentheses are free energies (kJ mol^{-1}) with respect to the starting energy of the three separated reactants FUR, AM and FA.

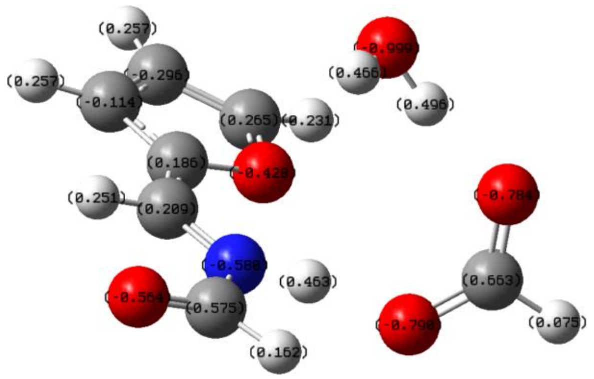

Supplementary Figure 58 NBO analysis on HCOO fragment: The total charge of HCOO fragment is -0.836 e, indicative of a $HCOO^-$ anion.

Supplementary References:

1. Kim, U. J. & Kuga, S. Thermal decomposition of dialdehyde cellulose and its nitrogen-containing derivatives, *Thermochim. Acta* **369**, 79-85 (2001).
2. Mou, K., Li, J., Wang, Y., Cha, R. & Jiang, X. 2,3-Dialdehyde nanofibrillated cellulose as a potential material for the treatment of MRSA infection, *J. Mater. Chem. B* **5**, 7876-7884 (2017).
3. Sivaraman, B., Sekhar, B. R., Nair, B. G., Hatode, V. & Mason, N. J. Infrared spectrum of formamide in the solid phase. *Spectrochim. Acta Part A: Mol. Biomol. Spectrosc.* **105**, 238-244 (2013).
