# Supplementary material for: N-formyl-stabilizing quasi-catalytic species afford rapid and selective solvent-free amination of biomass-derived feedstocks
Source: Nat Commun. 2019 Feb 11;10:699. doi: 10.1038/s41467-019-08577-4 (PMC6370847; doi:10.1038/s41467-019-08577-4)
Supplement: Supplementary file 3 — Supplementary Data 1 [file 41467_2019_8577_MOESM3_ESM.docx]

**Supplementary Data**

**FUR**

C -1.85904800 -0.55286000 -0.00003700

C -1.77545700 0.81255100 0.00014300

C -0.39084700 1.12335900 -0.00007500

C 0.27995600 -0.07536000 -0.00000500

H -2.69086900 -1.23797100 -0.00004300

H -2.60434700 1.50198500 0.00026900

H 0.06484300 2.10073100 -0.00011600

O -0.62973600 -1.10898000 -0.00001300

C 1.67795500 -0.44425700 0.00000200

H 1.86426700 -1.53334000 0.00001900

O 2.60108000 0.35747900 -0.00002300

**FA**

C 0.00000000 0.42076800 0.00000000

H -0.36639100 1.45346100 0.00000000

O 1.16286000 0.10348900 0.00000000

O -1.03058300 -0.43272400 0.00000000

H -0.69182400 -1.34419000 0.00000000

**AM**

C -0.15053300 0.38714100 -0.00003100

H -0.13070300 1.48852900 -0.00000200

O -1.20190000 -0.24351200 0.00000900

N 1.07885000 -0.15988100 0.00004400

H 1.19330800 -1.16375900 -0.00014100

H 1.90384300 0.41964500 -0.00005300

**FUR+AM+FA, no interaction**

C 7.73402800 -5.41397000 0.01741600

H 8.10407300 -6.45066900 0.05949900

O 6.53846800 -5.15375400 -0.06016700

N 8.72234900 -4.50182100 0.06008900

H 8.51041000 -3.51441700 0.02869900

H 9.68748700 -4.78618600 0.12419200

C 4.66259700 7.15544600 -0.00803300

H 5.19971700 8.11043400 0.00599300

O 5.18812200 6.07105700 -0.03689700

O 3.34200000 7.37036500 0.01154700

H 2.87804300 6.51590100 -0.00119200

C -7.44609900 0.13427700 0.04030200

C -6.36245700 0.96915800 0.04574800

C -5.21794700 0.13077600 0.01480300

C -5.68000200 -1.16264100 -0.00714800

H -8.50915800 0.31022700 0.05612000

H -6.38802700 2.04674200 0.06889700

H -4.18200200 0.43027200 0.00927700

O -7.05682100 -1.15721000 0.00864500

C -5.03876500 -2.45805700 -0.04177500

H -5.73676700 -3.31450600 -0.05187300

O -3.82781200 -2.62608100 -0.05914200

**FUR+AM+FA, with interaction**

C -4.21410200 0.46906800 -0.09274500

C -4.39526200 -0.88897400 -0.07223300

C -2.23209800 -0.56580800 0.02527500

H -4.99025100 1.21518500 -0.14611000

H -5.27497400 -1.51094400 -0.10103400

C -0.88098500 -1.04175100 0.10122300

H -0.77502800 -2.13700100 0.12784900

O 0.12199800 -0.32640900 0.13646900

C 3.43787500 -1.61505800 -0.01204700

H 2.33921700 -1.57997900 0.04660600

O 4.05913000 -2.67705300 -0.00916300

N 3.99228400 -0.39389300 -0.08430000

H 0.29824900 1.35398100 0.11870500

H 4.99695900 -0.30075000 -0.14150800

C 1.50995300 2.85764600 0.00495600

H 1.48107500 3.95382100 0.00594700

O 0.29469700 2.35215900 0.12119400

O 2.53964400 2.22257500 -0.09200800

H 3.42038600 0.44545300 -0.08207700

O -3.21558900 -1.53134600 -0.00158200

C -2.81632300 0.68045100 -0.02989800

H -2.29500400 1.62463600 -0.02474100

**TS1**

C -3.21823900 0.26193400 -0.35724100

C -3.04325600 -1.06827600 -0.58669400

C -1.21519200 -0.37037200 0.40466100

H -4.08861600 0.84557300 -0.61035000

H -3.65551200 -1.83061500 -1.03845700

C 0.11059400 -0.59981800 0.99176500

H 0.21313100 -1.58141200 1.45132800

O 0.68707000 0.36314100 1.68406600

C 2.41668400 -1.48761900 -0.10098600

H 3.10432100 -0.78354100 0.38323800

O 2.65579200 -2.65120100 -0.28108700

N 1.16704100 -0.89746500 -0.46270600

H 0.64099300 1.30387000 1.21719600

H 0.65121200 -1.50739200 -1.09827200

C 1.14431600 2.63003000 -0.61682400

H 1.28176900 3.63131200 -1.06228500

O 0.71916100 2.60710400 0.57464100

O 1.41867200 1.63933700 -1.33573800

H 1.26793000 0.09669600 -0.84462400

O -1.82966600 -1.47482300 -0.12704000

C -2.02670300 0.72041700 0.28347500

H -1.80197000 1.72153000 0.61348000

***N*-formyl carbinolamine + FA**

C 2.07528700 -1.86538600 -0.74596500

C 3.16770800 -1.22895200 -0.24989800

C 2.68260800 -0.04338900 0.39663600

C 1.33145400 -0.04946900 0.24312600

H 1.93165300 -2.77805800 -1.29932500

H 4.19153800 -1.55741900 -0.33168500

H 3.26286600 0.71040400 0.90508500

O 0.94280900 -1.15906400 -0.45596000

C 0.26890800 0.90047400 0.67723100

H 0.74932700 1.76900200 1.12636300

O -0.55841800 0.23211200 1.65502000

C -0.68243100 2.68661800 -0.73612500

H -1.40483600 2.84870900 -1.55040300

O -0.07320100 3.61009600 -0.20984500

N -0.55601500 1.37678800 -0.41559600

H -1.15750400 0.69428000 -0.87417500

H -1.09253200 0.89521700 2.11390100

C -2.68682300 -1.75736800 -0.30233400

H -3.36664400 -2.58952100 -0.51948700

O -2.43202000 -0.87960900 -1.10073000

O -2.20050100 -1.85220700 0.92542900

H -1.59137700 -1.09593300 1.13155600

**TS2**

C -2.81930400 0.73569400 -0.78351600

C -3.48489800 -0.14806600 0.01744700

C -2.49397100 -1.00441800 0.56717800

C -1.29206700 -0.58338700 0.05731100

H -3.14151200 1.55533600 -1.40411200

H -4.54896300 -0.18212900 0.18553400

H -2.63963400 -1.82844100 1.24788600

O -1.48917100 0.48846200 -0.77249000

C 0.03803300 -1.08663500 0.24950900

H 0.09680700 -2.07067800 0.69643000

O 0.56737500 -0.17465900 1.81745400

C 2.12424600 -1.63375300 -0.79040800

H 2.86315800 -1.22321900 -1.49090000

O 2.25992500 -2.69735500 -0.22336300

N 1.01908300 -0.80900900 -0.65636200

H 1.10021700 0.16731800 -1.01669100

H 1.32926900 -0.62916800 2.20593600

C 1.57224200 2.51556000 -0.02767500

H 1.90324300 3.56914300 -0.09699500

O 1.51957600 1.85726900 -1.08782600

O 1.28546300 2.11974200 1.14492100

H 0.89280400 0.78726500 1.49880400

**IM1**

C 2.60431400 -1.43659700 -0.37427100

C 3.58158900 -0.59557800 0.11242600

C 2.95611200 0.64229200 0.31570100

C 1.62405400 0.48374300 -0.06120200

H 2.62155200 -2.47348200 -0.67045600

H 4.61145500 -0.85523700 0.29330500

H 3.39661800 1.55338400 0.69014600

O 1.42710500 -0.81117100 -0.48601200

C 0.58045000 1.40035500 -0.05242800

H 0.80577100 2.40463400 0.29317700

O -1.28468600 -0.77999700 2.57734900

C -1.63234000 2.18963900 -0.36755500

H -2.61516900 1.83750000 -0.70225900

O -1.40340100 3.31290900 0.00333200

N -0.66320500 1.16289200 -0.42476500

H -1.02587800 0.19760400 -0.78254600

H -1.76820700 0.04927400 2.64565200

C -2.23296100 -1.95913800 -0.82620700

H -2.70975000 -2.72903500 -1.46616500

O -1.73609300 -0.97334100 -1.44736400

O -2.24522900 -2.15643300 0.40712800

H -1.62282700 -1.20891700 1.75825000

**TS3**

C 3.09564000 -1.46869300 -0.87098000

C 3.12358600 -1.98319400 0.39708300

C 2.22959800 -1.19768300 1.16306200

C 1.71793400 -0.24567200 0.30753800

H 3.60679000 -1.72994100 -1.78280100

H 3.71651700 -2.81810800 0.73281000

H 1.98765800 -1.30371700 2.20879900

O 2.24821000 -0.41805500 -0.94618800

C 0.77171100 0.79289900 0.50198300

H 0.48008300 1.02797400 1.51841700

C -0.13265300 2.93208000 -0.14739400

H -0.16162200 3.61717900 -1.00304200

O -0.69543100 3.12494700 0.90384900

N 0.64931900 1.80551800 -0.39636700

H 1.04593000 1.69632600 -1.32319000

C -1.72872400 -0.20760400 -0.13826200

H -0.61836500 0.05049600 0.28552100

O -2.21609800 -1.16767100 0.46349600

O -2.05347100 0.54573800 -1.04443900

O -4.76171700 -2.27231200 0.00281900

H -3.89290000 -1.84235200 0.12992400

H -5.34381300 -1.57045200 -0.30635900

**1+CO_2_+H_2_O**

C -2.35271800 -2.44580300 0.34138300

C -3.38441600 -2.01285600 -0.42792600

C -3.00714300 -0.71417700 -0.91035200

C -1.77253000 -0.45229500 -0.40120100

H -2.17114900 -3.34377600 0.90769400

H -4.29990100 -2.54642800 -0.62935500

H -3.58027000 -0.06199800 -1.55057100

O -1.35749000 -1.50964000 0.36761900

C -0.83806000 0.70609600 -0.51539500

H -1.20607100 1.37539900 -1.29204200

C -1.44787300 2.55992200 1.01565900

H -1.19295300 2.99843600 1.99319600

O -2.31967000 3.03462300 0.29415000

N -0.69618700 1.48006500 0.72124500

H -0.02199700 1.17456600 1.40824500

C 3.81308600 0.14599300 -1.36793800

H 0.15682300 0.36261200 -0.80714700

O 4.07124300 0.20060700 -0.23621200

O 3.55759400 0.09460900 -2.49697500

O 3.85332300 -1.18955200 2.56726200

H 4.76633200 -1.32395000 2.84419800

H 3.92041500 -0.76178600 1.70494300

***N*-Formyl imine + AM**

C -2.43828100 -1.77389400 0.20260100

C -3.55668200 -1.02506800 -0.04216300

C -3.10163200 0.30272200 -0.23343500

C -1.73029800 0.27692100 -0.09034500

H -2.28762100 -2.82212700 0.40290600

H -4.57132800 -1.38651200 -0.07985500

H -3.69520500 1.17746400 -0.44884600

O -1.32581200 -1.00871300 0.17849900

C -0.77313900 1.32312100 -0.17212100

H -1.18830900 2.31497000 -0.36611300

C 3.17388200 -1.86482800 -0.02143500

H 3.44668500 -2.93054400 0.05484600

O 4.02579900 -0.99523000 -0.19447800

N 1.85495200 -1.64708700 0.09342200

H 1.22808700 -2.42433100 0.23256800

C 1.30965900 2.28641100 -0.04927200

H 2.32576400 2.08849600 -0.41975600

O 0.98247500 3.38546200 0.35818800

N 0.50593500 1.14505500 -0.05308400

H 1.45495700 -0.70766500 0.04122900

**TS4**

C -2.94918600 -0.45770400 -0.66687200

C -3.07609900 -0.58860400 0.67965900

C -1.76107000 -0.42144000 1.22426100

C -0.93241200 -0.19492000 0.16795300

H -3.64430400 -0.50527500 -1.48818300

H -3.98924500 -0.77970600 1.22046800

H -1.47108000 -0.46064000 2.26245600

O -1.64867000 -0.21388800 -1.00008300

C 0.52614600 0.05453400 0.07592100

H 0.95277400 0.07597100 1.07971500

C 2.31300900 -1.81423400 -0.11532000

H 2.60914800 -2.67303200 -0.73082900

O 2.81472800 -1.50405600 0.92388500

N 1.26075000 -1.03417500 -0.73157600

H 0.65246500 -1.60773400 -1.31843200

C 1.07721700 2.37571500 -0.31483200

H 1.55718800 3.07255600 -1.02371800

O 0.67760800 2.78265800 0.79228300

N 0.99256100 1.13683600 -0.79579100

H 1.62492100 -0.07856100 -1.34319400

**FDFAM**

C -3.13125000 -0.27992200 -0.55491800

C -3.23366600 0.10506400 0.74269800

C -1.89045600 0.24946400 1.22916700

C -1.07204600 -0.05810600 0.18890200

H -3.84872300 -0.51049700 -1.32408000

H -4.14797600 0.26663800 1.29118300

H -1.57980100 0.54277000 2.21951300

O -1.81565900 -0.38488400 -0.91404700

C 0.41346400 -0.09079600 0.03182000

H 0.86696600 0.15338400 0.99114400

C 2.04521600 -1.94962300 0.03083000

H 2.20302100 -2.96482600 -0.36372200

O 2.86150300 -1.38579100 0.74624000

N 0.85945700 -1.42221800 -0.35808200

H 0.26607800 -1.97309800 -0.96307800

C 1.56900400 2.03268700 -0.53870100

H 1.84333000 2.66465900 -1.39730100

O 1.85719000 2.33080300 0.61354800

N 0.89605400 0.92021800 -0.91384400

H 0.68266800 0.80134300 -1.89490200

**FDFAM+FA**

C 1.82843800 -2.72218100 -0.35834200

C 3.04599600 -2.23089300 -0.01364700

C 2.87976000 -0.81350700 0.14591800

C 1.57183100 -0.54992000 -0.11273100

H 1.45714200 -3.70786000 -0.58234100

H 3.95296500 -2.80071500 0.11150100

H 3.63369100 -0.09124200 0.41642700

O 0.91160900 -1.70922800 -0.42321400

C 0.76274300 0.70698400 -0.10983000

H 1.43109900 1.54306800 0.09029800

C -0.06672000 2.14668800 -1.94318800

H -0.53565300 2.10175600 -2.93799300

O 0.22646800 3.20339100 -1.40054600

N 0.14666400 0.91823100 -1.41511300

H -0.14512600 0.11047100 -1.94800600

C -0.13458600 1.50070400 2.05196600

H -0.98986400 1.38092700 2.73485800

O 0.79316600 2.26529800 2.29732800

N -0.24427900 0.72390600 0.95300700

H -1.04783700 0.10352100 0.87966700

H -3.21061600 -0.46789300 -1.20676500

C -3.62458400 -1.43840100 0.36823100

H -4.39763200 -2.11435500 0.74806600

O -3.90287700 -1.06828300 -0.88145700

O -2.65863300 -1.08486400 1.00273600

**TS5**

C -3.33439900 0.28606300 -0.02679200

C -3.56644800 -1.05365500 -0.14086500

C -2.30953600 -1.69049600 0.05430800

C -1.40109500 -0.68903900 0.28058600

H -3.96946700 1.15384500 -0.09092100

H -4.51676000 -1.52433500 -0.33337800

H -2.09829300 -2.74833200 0.03940200

O -2.02522900 0.52737800 0.22691000

C 0.01680600 -0.74233400 0.55641900

H 0.37034000 -1.73060200 0.82627000

C 2.10636800 -1.25984500 -1.21669400

H 2.89982300 -0.64535300 -0.77392900

O 2.28807500 -2.35537100 -1.68273600

N 0.82160000 -0.65603800 -1.16072900

H 0.17132400 -1.13887600 -1.78042800

C 1.75118000 0.04433700 2.00281900

H 2.12116600 0.95293900 2.49514200

O 2.29865000 -1.03766300 2.10010500

N 0.60009000 0.27237800 1.28043600

H 0.35690900 1.27127800 1.06253300

H 0.82688000 0.40659100 -1.32176400

C 0.61798100 2.93441300 -0.78593400

H 0.67845600 3.95458900 -1.21492800

O 0.83223000 1.98511300 -1.59168400

O 0.34660400 2.85019400 0.43627400

**IM2**

C -0.63208200 3.09799700 -0.98127600

C -1.62128400 3.35409600 -0.05817100

C -1.46079400 2.39443400 0.95292100

C -0.37540200 1.60431000 0.58502700

H -0.36884600 3.57225100 -1.91337600

H -2.35967800 4.13647700 -0.11724200

H -2.04940900 2.27264100 1.84895900

O 0.12425600 2.05564000 -0.61535500

C 0.19932300 0.51791700 1.23874300

H -0.25028100 0.21758100 2.18124300

C -3.82638600 -2.39967000 -0.81853700

H -4.46654400 -3.09298600 -1.38685600

O -4.28549000 -1.62666600 0.01516800

N -2.52445200 -2.50266600 -1.14217500

H -2.21366600 -3.16470300 -1.83617300

C 1.71866400 -1.26489900 1.60010500

H 2.58395800 -1.73441400 1.11588600

O 1.22627900 -1.60748400 2.64724600

N 1.23640300 -0.17948400 0.82423000

H 1.79364700 0.01107800 -0.11503500

H -1.83558800 -1.92193100 -0.68506600

C 3.57038200 -0.66173900 -1.51237800

H 4.16262500 -0.50852500 -2.43886100

O 3.88896400 -1.59944100 -0.76020400

O 2.61919200 0.16956500 -1.34516400

***N*-Formyl imine + AM**

C 2.66586100 -1.97145000 0.34655300

C 3.89211000 -1.48400900 -0.01484700

C 3.70233600 -0.10386300 -0.27058400

C 2.36699400 0.16078400 -0.04970700

H 2.32169300 -2.95269500 0.63007100

H 4.80769800 -2.04818300 -0.08519800

H 4.44340900 0.61666800 -0.57982700

O 1.73131200 -0.99672400 0.33190100

C 1.63410700 1.37093900 -0.16514600

H 2.21598600 2.23481900 -0.49408400

C -2.39734800 -0.36414600 2.18072700

H -2.74968800 -1.17233500 2.84177800

O -2.97427900 0.70970400 2.10985000

N -1.30880700 -0.70965300 1.43605000

H -0.77007100 -1.50399600 1.75754900

C -0.23150500 2.71458300 -0.14557900

H -1.11192000 2.89256200 0.48816300

O 0.11291600 3.51065500 -0.99874400

N 0.37160900 1.48080400 0.11298900

H -0.76597600 0.03987600 0.98755300

C -2.74571800 -1.42726900 -2.00748400

H -3.00233800 -2.07599500 -2.85408000

O -2.92373500 -0.23261200 -1.99053100

O -2.21712400 -2.14546300 -1.01791600

H -1.98215000 -1.56666000 -0.25527900

**TS4’**

C -3.28852600 1.34172200 0.15495300

C -3.50912900 0.02771700 -0.13693800

C -2.24520200 -0.52046100 -0.49424000

C -1.34234600 0.50554700 -0.40548200

H -3.93125200 2.14813400 0.46647900

H -4.45708800 -0.48423800 -0.10301900

H -2.02373500 -1.53719100 -0.77676000

O -1.97877800 1.65321800 -0.00197700

C 0.08289400 0.62572800 -0.62601800

H 0.42442900 1.61128500 -0.92285500

C 2.08053200 1.24564800 1.22428400

H 2.88174700 0.57387600 0.89305400

O 2.26390200 2.37275300 1.60531200

N 0.78286200 0.67184500 1.13264000

H 0.11126100 1.21951900 1.67116400

C 1.91211500 -0.19398000 -1.93353600

H 2.31882400 -1.11626000 -2.36804100

O 2.45441100 0.88949500 -2.03966100

N 0.72428900 -0.40646600 -1.26834600

H 0.45459200 -1.39258000 -1.02530600

C 0.37282300 -2.91802900 0.95068900

H 0.33065500 -3.91500300 1.43195200

O 0.14896500 -2.87799500 -0.28553700

O 0.63915400 -1.94695600 1.71099000

H 0.74030300 -0.37266200 1.36799700

**IM0a, FUR+AM**

C 3.27837200 1.00548100 -0.00129400

C 3.64602000 -0.31304100 -0.00224100

C 2.43987400 -1.05825000 -0.00067600

C 1.41325600 -0.14401000 0.00094500

H 3.84183200 1.92414800 -0.00181800

H 4.65465800 -0.69366800 -0.00379000

H 2.32817600 -2.13083400 -0.00078700

O 1.93637600 1.13037900 0.00058900

C -0.02347900 -0.25069000 0.00285700

H -0.57192000 0.70633700 0.00368300

O -0.63026300 -1.31856100 0.00335100

C -3.91070100 0.46701700 -0.00181500

H -5.00106200 0.62654900 -0.00464700

O -3.12210600 1.41046400 0.00146200

N -3.56181500 -0.82924900 -0.00242700

H -2.57731500 -1.10174100 -0.00019800

H -4.27376800 -1.54334400 -0.00532600

**TS^**

C 2.85193000 -0.73603400 -0.08224600

C 2.90737500 0.37794900 0.69648200

C 1.62172400 1.00079800 0.59868000

C 0.87824400 0.22069300 -0.23535900

H 3.56096100 -1.51017500 -0.32363900

H 3.75772200 0.71652500 1.26701300

H 1.29250400 1.90852100 1.07777800

O 1.62373900 -0.84773300 -0.66232300

C -0.50704400 0.32083400 -0.74877600

H -0.62832300 -0.19007300 -1.71486000

O -1.16159100 1.40210600 -0.52370800

C -2.61288600 -0.82564100 0.53375700

H -3.18806000 -1.56396200 1.09146300

O -3.25640400 0.27673300 0.24163400

N -1.39227400 -1.02422800 0.18515600

H -0.97206900 -1.90524200 0.46087400

H -2.55883100 0.91356300 -0.17477800

**IM0b, *N*-formyl carbinolamine + FA**

C -2.92997600 -0.40522800 -0.56390300

C -2.94829800 0.66922500 0.26791500

C -1.58728400 0.90191700 0.65303100

C -0.84279600 -0.04863300 0.02415400

H -3.69159400 -0.94441900 -1.10144100

H -3.81837200 1.22914000 0.57189700

H -1.22016900 1.67110400 1.31446000

O -1.65349100 -0.85706700 -0.72444500

C 0.60907800 -0.38929400 0.01987100

H 0.78851600 -1.14974100 -0.74585800

O 0.97427900 -0.91191200 1.29386400

C 2.75299800 0.69115400 -0.44873100

H 3.23863700 1.63611300 -0.73117600

O 3.38171600 -0.34920300 -0.26744400

N 1.41414500 0.78673000 -0.31454300

H 1.89593800 -1.20047800 1.21273300

H 0.96566800 1.68178600 -0.44864500

**TS^^**

C 2.80004000 -0.18029300 -0.54067500

C 2.61455700 -0.82777300 0.64247800

C 1.23657200 -0.65620800 0.98008700

C 0.68378000 0.08316200 -0.02406500

H 3.65398800 -0.03821700 -1.18178800

H 3.36405200 -1.36419300 1.20204300

H 0.71854300 -1.03232300 1.84724300

O 1.63630600 0.37967900 -0.96546100

C -0.66856300 0.58933400 -0.26524400

H -0.91450600 0.66854600 -1.32576700

O -0.78001900 2.07887700 0.31920400

C -2.58608600 -0.77156800 0.17526500

H -3.44097700 -0.89191400 0.86078500

O -2.50263400 -1.45617500 -0.85585900

N -1.70721700 0.14017900 0.60914700

H -1.61193800 1.53905600 0.88682900

H -1.12966400 2.69882800 -0.34352600

**IM^**

C -3.50656300 0.87530000 0.04849300

C -3.65988900 -0.46634400 0.27852000

C -2.36394500 -1.03004800 0.19984200

C -1.49802400 0.00631200 -0.07303900

H -4.20241600 1.69725600 0.00977200

H -4.58833700 -0.97668200 0.47751000

H -2.08626300 -2.06452900 0.32325600

O -2.21237500 1.18125800 -0.16666800

C -0.09000500 0.10408100 -0.26072100

H 0.30088500 1.10878100 -0.44475500

O 5.47868100 0.43537900 0.28221400

C 2.05160600 -0.73034300 -0.28863700

H 2.59141700 -1.52124100 -0.82744100

O 2.65123500 0.18509600 0.26153400

N 0.68817600 -0.92997800 -0.24618800

H 5.72074600 0.94480800 -0.49812700

H 4.50733000 0.35383900 0.23971000

**FUR+NH_3_**

C 2.57053600 -0.83193500 0.01368600

C 2.88987000 0.49690100 0.00246600

C 1.65318500 1.19436000 -0.01012600

C 0.66378100 0.23954700 -0.00565800

H 3.16690600 -1.72961600 0.02504500

H 3.88315700 0.91556100 0.00318400

H 1.50005700 2.26238200 -0.02103500

O 1.23082300 -1.00961800 0.00904700

C -0.77159400 0.37225500 -0.01376600

H -1.11614100 1.42081600 -0.02570100

O -1.56950100 -0.55640400 -0.00849200

N -4.68718200 0.18946300 -0.02880100

H -5.18489200 -0.47803900 -0.61115400

H -5.05408400 0.08505200 0.91294400

H -3.70997300 -0.10098900 -0.00572400

**TS^#^**

C -1.99458500 -0.90568700 -0.04220800

C -2.39708600 0.38579400 0.07872900

C -1.20011300 1.17753300 0.10021600

C -0.15826600 0.30841100 -0.01264000

H -2.51615300 -1.84641600 -0.09817200

H -3.41554000 0.73482800 0.14350600

H -1.12597400 2.25036000 0.18625500

O -0.63151000 -0.97389900 -0.10201400

C 1.31869600 0.50958100 -0.09132100

H 1.52161000 1.57669000 0.07019600

O 1.96902200 -0.05144000 -1.18179200

H 2.55108400 -0.64101800 -0.11285200

N 2.09404700 -0.29014800 0.96925100

H 2.66977000 0.26665200 1.59730900

H 1.54490600 -0.96113700 1.50280100

**Amino(furan-2-yl)methanol**

C -1.99170500 -0.86861600 -0.14846300

C -2.35771400 0.41768500 0.08636500

C -1.13749100 1.16545500 0.20690800

C -0.12060100 0.27807900 0.03523900

H -2.53938000 -1.78237300 -0.30705300

H -3.36636900 0.79157200 0.16487200

H -1.03342200 2.22235000 0.39685600

O -0.62935400 -0.97434100 -0.18302500

C 1.36972100 0.41756600 0.08112700

H 1.58786500 1.45446900 0.35826500

O 1.86920700 0.14090500 -1.23087000

H 2.82725100 0.05187100 -1.14234000

N 2.05660100 -0.43960400 1.03779300

H 1.96394800 -0.07402700 1.98030600

H 1.67182100 -1.38015600 1.02864300

**Supplementary Data** Cartesian coordinates of important intermediates at reaction temperature of 180 °C at the level of B3LYP/6-311+G(2s,2p).
